# ﻿A survey of the spider genus *Dysdera* Latreille, 1804 (Araneae, Dysderidae) in Iran, with fourteen new species and notes on two fossil genera

**DOI:** 10.3897/zookeys.1146.97517

**Published:** 2023-02-07

**Authors:** Alireza Zamani, Yuri M. Marusik, Tamás Szűts

**Affiliations:** 1 Zoological Museum, Biodiversity Unit, FI-20014 University of Turku, Turku, Finland University of Turku Turku Finland; 2 Department of Zoology & Entomology, University of the Free State, Bloemfontein 9300, South Africa University of the Free State Bloemfontein South Africa; 3 Department of Ecology, University of Veterinary Medicine Budapest, Rottenbiller u. 50, Budapest, 1077, Hungary University of Veterinary Medicine Budapest Budapest Hungary

**Keywords:** Aranei, Middle East, *Mistura*, red devil spiders, *Segistriites*, woodlouse hunters

## Abstract

The taxonomy of the Iranian species of the dysderid spider genus *Dysdera* Latreille, 1804 is revised. Currently, the only species of this genus known from Iran is *D.pococki* Dunin, 1985, albeit on the basis of a doubtful record. The following 14 species are described as new to science in this paper: *D.achaemenes***sp. nov.** (♀; Fars), *D.bakhtiari***sp. nov.** (♂; Chaharmahal & Bakhtiari), *D.damavandica***sp. nov.** (♂; Mazandaran), *D.genoensis***sp. nov.** (♂♀; Hormozgan), *D.hormuzensis***sp. nov.** (♀; Hormozgan), *D.iranica***sp. nov.** (♂♀; Fars, Hormozgan), *D.isfahanica***sp. nov.** (♂♀; Isfahan), *D.mazeruni***sp. nov.** (♀; Mazandaran), *D.medes***sp. nov.** (♂; Tehran), *D.persica***sp. nov.** (♂♀; Golestan, Mazandaran), *D.sagartia***sp. nov.** (♂♀; Tehran), *D.tapuria***sp. nov.** (♂♀; Mazandaran), *D.verkana***sp. nov.** (♂; Golestan), and *D.xerxesi***sp. nov.** (♂; Bushehr). Distribution records of all species are mapped. Also, the taxonomy of *Mistura* Petrunkevitch, 1971 and *Segistriites* Straus, 1967, two fossil genera currently considered in Dysderidae, is discussed and the latter is transferred to Segestriidae.

## ﻿Introduction

The spider family Dysderidae C.L. Koch, 1837 comprises 591 extant species in 25 genera distributed in the West Palaearctic (WSC 2022). Most species have limited dispersal abilities and very small ranges; one exception is *Dysderacrocata* C.L. Koch, 1838 which has a cosmopolitan distribution due to anthropogenic transportations (Jocqué and Dippenaar-Schoeman 2006).

Although the first record of this family in Iran dates back to late 19^th^ century (Pocock 1889), the dysderid fauna of this country remains almost completely unknown. Currently, there are only three species of Dysderidae known from Iran: *Dysderapococki* Dunin, 1985, *Dysderellatranscaspica* (Dunin & Fet, 1985), and *Harpacteaparthica* Brignoli, 1980 (Zamani et al. 2022a). The Iranian records of several species (i.e., *Dysderaaculeata* Kroneberg, 1875, *D.asiatica* Nosek, 1905, *D.erythrina* (Walckenaer, 1802), *Harpacteababori* (Nosek, 1905), *H.dobati* Alicata, 1974, and *Tediaoxygnatha* Simon, 1882) were recently considered as misidentifications and subsequently these species were rejected from the checklist of Iranian spiders (Zamani et al. 2017, 2022b). Recently, we had the opportunity to examine a collection of Iranian specimens of *Dysdera* Latreille, 1804, in which 14 species new to science were detected. In this paper, all species of this genus occurring in Iran are surveyed, their distributions are mapped, and those new to science are described and illustrated. Additionally, the taxonomy of two fossil genera currently considered in Dysderidae is discussed, and one of them is herein transferred to Segestriidae.

## ﻿Materials and methods

Photographs of specimens and their copulatory organs were obtained using a Nikon D300S DSLR camera attached to a Nikon S800 stereomicroscope, a Tucsen TrueChrome Metrics microscope camera attached to a Nikon Eclipse E200 compound microscope, and an Olympus Camedia E‐520 camera attached to an Olympus SZX16 stereomicroscope or to the eye piece of an Olympus BH2 transmission microscope. Digital images of different focal planes were stacked with Helicon Focus™ 8.1.1. Illustrations of internal genitalia were made after digesting tissues off with Neo PanPur commercial pancreatic enzyme cocktail pill, clearing the structures in wintergreen oil (methyl-salicylate), then mounting them on a temperate slide preparation (Coddington 1983). Body measurements exclude the chelicerae and spinnerets. Leg segments were measured on the dorsal side. Measurements of legs are listed as: total length (femur, patella, tibia, metatarsus, tarsus). All measurements are given in millimetres. Geographic coordinates of collection localities were obtained from the labels or georeferenced using Google Earth. Measurements and characters of the palp used in the diagnoses are based on the retrolateral view, unless otherwise indicated.

**Abbreviations**: Eyes: **AME** ‒ anterior median eye, **PLE** ‒ posterior lateral eye, **PME** ‒ posterior median eye. Spination: **d** ‒ dorsal, **Fe** ‒ femur, **Mt** ‒ metatarsus, **Pa** ‒ patella, **pl** ‒ prolateral, **rl** ‒ retrolateral, **Ti** ‒ tibia, **v** ‒ ventral.

**Depositories: MHNG** – Muséum d’histoire naturelle, Genève, Switzerland (P.J. Schwendinger, L. Monod); **MMUE** – Manchester Museum of the University of Manchester, United Kingdom (D.V. Logunov); **SMF** – Senckenberg Museum, Frankfurt am Main, Germany (P. Jäger); **ZMUT** – Zoological Museum of the University of Turku, Finland (V. Vahtera).

## ﻿Taxonomy

### ﻿Family Dysderidae

Taxon classificationAnimaliaAraneaeDysderidae

C.L. Koch, 1837

8753AB6B-107F-5E81-AD07-095A03823F6C

#### Comments.

The family was divided into four tribes (i.e., Dysderini, Harpactini, Orsolobini and Rhodini) by Cooke (1965), of which three were elevated to subfamilies (i.e., Dysderinae, Harpacteinae and Rhodinae) by Deeleman-Reinhold and Deeleman (1988), and one was elevated to the family-level (i.e., Orsolobidae Cooke, 1965) by Forster and Platnick (1985).

Although Dysderidae appears to be a monophyletic family often considered restricted to the Palaearctic, it is in fact distributed only in the West Palaearctic (from Canary Islands to west Xinjiang) and polyphyletic with its current generic composition. Eleven species of five genera are known from fossils (Dunlop et al. 2020): *Dasumiana* Wunderlich, 2004 (3 spp.), *Dysdera* (1 sp.), *Harpactea* Bristowe, 1939 (5 spp.), *Segistriites* Straus, 1967 (1 sp.), and *Mistura* Petrunkevitch, 1971 (1 sp.). Judging by the position of the legs (i.e., legs I–III directed forwards) and the overall somatic features of *Segistriitescromei* Straus, 1967, this monotypic Neogene fossil genus is herein transferred to Segestriidae Simon, 1893. At the time of the description of *Segistriites*, Segestriidae was not a separate family but rather a subfamily (i.e., Segestriinae Simon, 1893) of Dysderidae. Furthermore, Strauss (1967) explicitly mentions the close affinity of this genus to *Segestria* Latreille, 1804, the type genus of Segestriidae. The monotypic Neogene fossil genus *Mistura* also appears to be misplaced in Dysderidae: the holotype specimen of *Misturaperplexa* Petrunkevitch, 1971 has an unknown arrangement of eyes and several characters different from Dysderidae, including a lack of claw tufts, the presence of an onychium, and long spinnerets (Petrunkevitch 1971).

#### Composition.

More than 600 species in 26 genera (Dunlop et al. 2020; WSC 2022; current paper).

### ﻿Subfamily Dysderinae

Taxon classificationAnimaliaAraneaeDysderidae

C.L. Koch, 1837

42DB2826-8091-5FE7-B12E-BB80F0F2CAE9

#### Diagnosis.

This subfamily can be diagnosed from other dysderids by the edge of sternum-labium joint ca. 2.5–3× longer than the edge of the maxilla-sternum joint, all tarsi bearing claw tufts, posterior metatarsi bearing scopulae, and the spineless anterior tibiae and metatarsi. Furthermore, the bulb of dysderines does not bear a free embolus (with the exception of *Harpactocrates* Simon, 1914), and the posterior diverticulum of endogyne is large and wide (Deeleman-Reinhold and Deeleman 1988; Le Peru 2011; Kunt et al. 2019).

#### Composition.

Around 360 species in 11 genera: *Cryptoparachtes* Dunin, 1992, *Dysdera* Latreille, 1804, *Dysderella* Dunin, 1992, *Dysderocrates* Deeleman-Reinhold & Deeleman, 1988, *Harpactocrates*, *Hygrocrates* Deeleman-Reinhold, 1988, *Kut* Kunt, Elverici, Yağmur & Özkütük, 2019, *Parachtes* Alicata, 1964, *Rhodera* Deeleman-Reinhold, 1989, *Stalitochara* Simon, 1913, and *Tedia* Simon, 1882. The position of *Rhodera* in Dysderinae is questionable (see Le Peru 2011).

### ﻿Genus Dysdera

Taxon classificationAnimaliaAraneaeDysderidae

Latreille, 1804

E880632E-356B-528F-934A-5208AE2CF592

#### Type species.

*Araneaerythrina* Walckenaer, 1802, from France.

#### Diagnosis.

*Dysdera* can be diagnosed from other dysderine genera by the interdistance of PLE and PME less than half of their diameter, three or four cheliceral teeth in one series, punctiform (= highly reduced) fovea, and femur I at least twice as long as coxa I. The bulb is cylindrical, bearing a broad posterior apophysis and a distal psembolus. The endogyne is composed by an anterior diverticulum bearing a dorsal arch and a ventral arch, a transverse receptacle and a posterior diverticulum bearing a transverse bar (Deeleman-Reinhold and Deeleman 1988; Le Peru 2011).

#### Comments.

Deeleman-Reinhold and Deeleman (1988) proposed nine species groups within *Dysdera*: *aculeata*, *asiatica*, *crocata*, *erythrina*, *festai*, *lata*, *longirostris*, *ninnii*, and *punctata*. Charitonov (1956) and Fomichev and Marusik (2021) proposed an additional *cylindrica* group composed of Central Asian species considered within *asiatica* group by Deeleman-Reinhold and Deeleman (1988), mostly based on their disjunct distribution. Here, we tentatively treat these species within *aculeata* group, primarily on the basis of the conformation of male palp and considering that the discovery of similar species in Iran fills this distributional gap. Furthermore, characters based on spination used by Deeleman-Reinhold and Deeleman (1988) in definition of the species groups are not followed here as they appear to be variable; assignment of the species treated here to their respective groups is primarily based on the conformation of male palp.

#### Composition.

More than 310 species (WSC 2022).

##### ﻿*aculeata* species group

**Diagnosis.** This group can be diagnosed by a combination of the following characters: the carapace elongated and hexagonal, and the psembolus longer than the tegulum, with an anterior (= median) crest and an acuminate apex (Deeleman-Reinhold and Deeleman 1988).

**Comments.** Currently, there is no clear distinction between the *aculeata* and *asiatica* groups, both of which are in serious need of a thorough revision (see Dimitrov 2021).

### ﻿ Dysdera achaemenes sp. nov.

Taxon classificationAnimaliaAraneaeDysderidae

645A6489-2988-521F-87AB-CADEBC6BAFDC

https://zoobank.org/83006184-7584-4AB0-9862-8C050A4A719C

Figs 1A–C , 2A, B

#### Type material.

***Holotype*** ♀ (ZMUT), Iran: Fars Province: Khanj, Khan Cave, 27°44'N, 53°20'E.

#### Etymology.

The specific epithet is a noun in apposition, referring to the apical ancestor of the Achaemenid dynasty of rulers of Persia.

#### Diagnosis.

The new species differs from its congeners occurring in the region by the very long receptacle (*Re*), longer than the posterior margin of the dorsal arch (*Da*) (vs. shorter).

#### Description.

**Figure 1.**
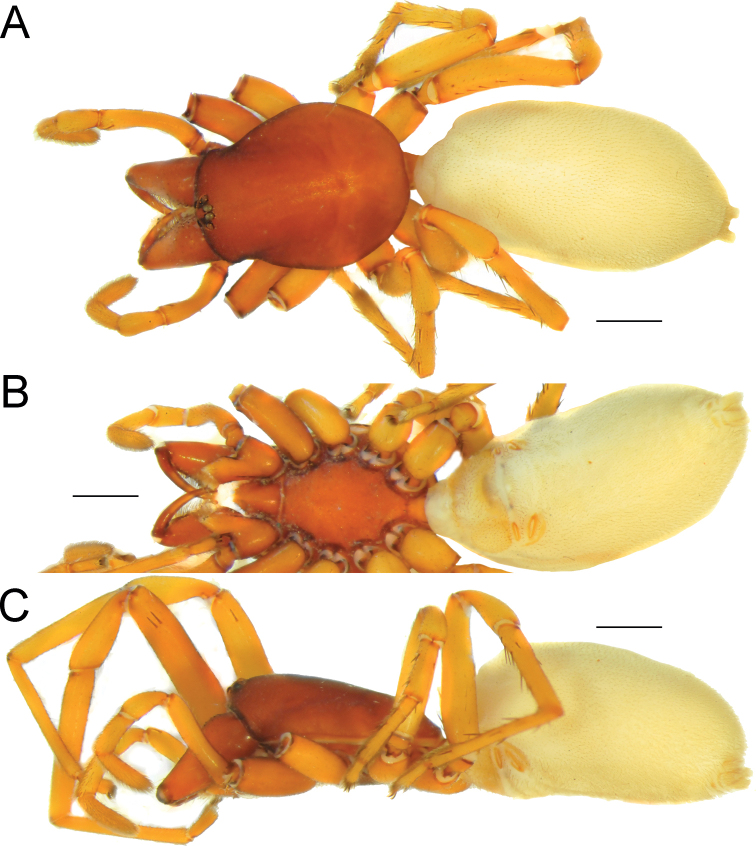
Female of *Dysderaachaemenes* sp. nov., habitus. **A** dorsal view **B** ventral view **C** lateral view. Scale bars: 1.0 mm.

**Female.** Habitus as in Fig. 1A–C. Total length 8.13. Carapace 3.28 long, 2.58 wide. Eye diameters: AME 0.12, PME 0.12, PLE 0.14. Carapace, sternum, chelicerae, labium, and maxillae reddish. Legs pale orange. Abdomen pale cream-coloured, without any pattern. Spinnerets uniformly pale cream-coloured. Measurements of legs: I: 8.77 (2.64, 1.56, 2.21, 1.78, 0.58), II: 8.49 (2.42, 1.44, 2.02, 2.08, 0.53), III: 6.54 (1.87, 0.99, 1.30, 1.92, 0.46), IV: 8.99 (2.51, 1.32, 2.07, 2.47, 0.62). Spination: I: Fe: 3pl. II: Fe: 1pl. III: Fe: 1pl; Ti: 4pl, 1rl, 6v; Mt: 3pl, 2rl, 6v. IV: Fe: 9d, 1rl; Ti: 4pl, 6v; Mt: 3pl, 2rl, 5v.

**Figure 2.**
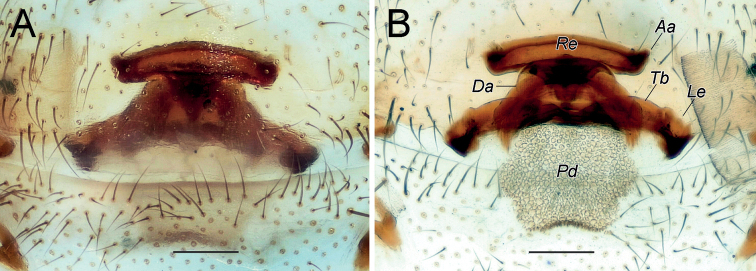
Female of *Dysderaachaemenes* sp. nov., endogyne. **A** ventral view **B** dorsal view. Scale bars: 0.25 mm. Abbreviations: *Aa* – anterior angle, *Da* – dorsal arch, *Le* – lateral edge, *Pd* – posterior diverticulum, *Re* – receptacle, *Tb* – transverse bar.

Endogyne as in Fig. 2A, B; length/width ratio ca. 2.6; receptacle slightly arched, 5× longer than wide, anterior angles (*Aa*) rounded, not long; dorsal arch (*Da*) trapezoidal; transverse bar (*Tb*) as wide as receptacle, lateral edges (Le) directed posterolaterally; posterior diverticulum (*Pd*) hexagonal.

**Male.** Unknown.

#### Distribution.

Known only from the type locality in Fars Province, southern Iran (Fig. 35).

### ﻿ Dysdera bakhtiari sp. nov.

Taxon classificationAnimaliaAraneaeDysderidae

D067C2D8-0FD4-5FE7-AE47-98F1868E023C

https://zoobank.org/8B8C7362-DAA2-4167-ACE9-4BEB6528ACBE

Figs 3 , 4A–D

#### Type material.

***Holotype*** ♂ (MHNG), Iran: Chaharmahal & Bakhtiari Province: Zard Kuh, 32°23'N, 50°07'E, 2700 m, 20.06.1974 (A. Senglet).

#### Etymology.

The specific epithet is a noun in apposition, referring to an Iranian tribe primarily inhabiting Chaharmahal & Bakhtiari, Khuzestan, Lorestan, Bushehr, and Isfahan provinces.

#### Diagnosis.

This species can be distinguished from other species of the *aculeata* group occurring in the region by having a wider psembolus (i.e., 1.5× wider than the tegulum).

#### Description.

**Figure 3.**
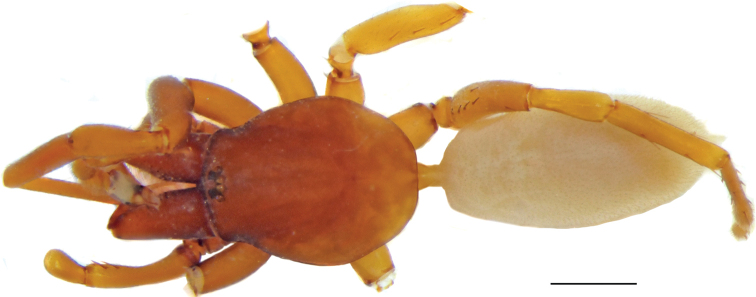
Male of *Dysderabakhtiari* sp. nov., habitus, dorsal view. Scale bar: 1.0 mm.

**Male.** Habitus as in Fig. 3. Total length 5.97. Carapace 2.55 long, 1.97 wide. Eye diameters: AME 0.10, PME 0.08, PLE 0.11. Carapace, sternum, chelicerae, labium, and maxillae reddish brown. Legs orange. Abdomen cream-coloured, without any pattern. Spinnerets uniformly cream-coloured. Measurements of legs: I: 7.40 (2.07, 1.26, 1.80, 1.82, 0.45), II: 6.68 (1.83, 1.17, 1.61, 1.61, 0.46), III: 5.02 (1.42, 0.81, 0.98, 1.40, 0.41), IV: 6.51 (1.87, 0.97, 1.43, 1.77, 0.47). Spination: I: Fe: 4pl. II: Fe: 3pl. III: Fe: 3pl, 1rl; Ti: 4pl, 2rl, 5v; Mt: 6pl, 2rl, 2v. IV: Fe: 7d, 1pl; Ti: 5pl, 2rl, 5v; Mt: 5pl, 2rl, 5v.

**Figure 4.**
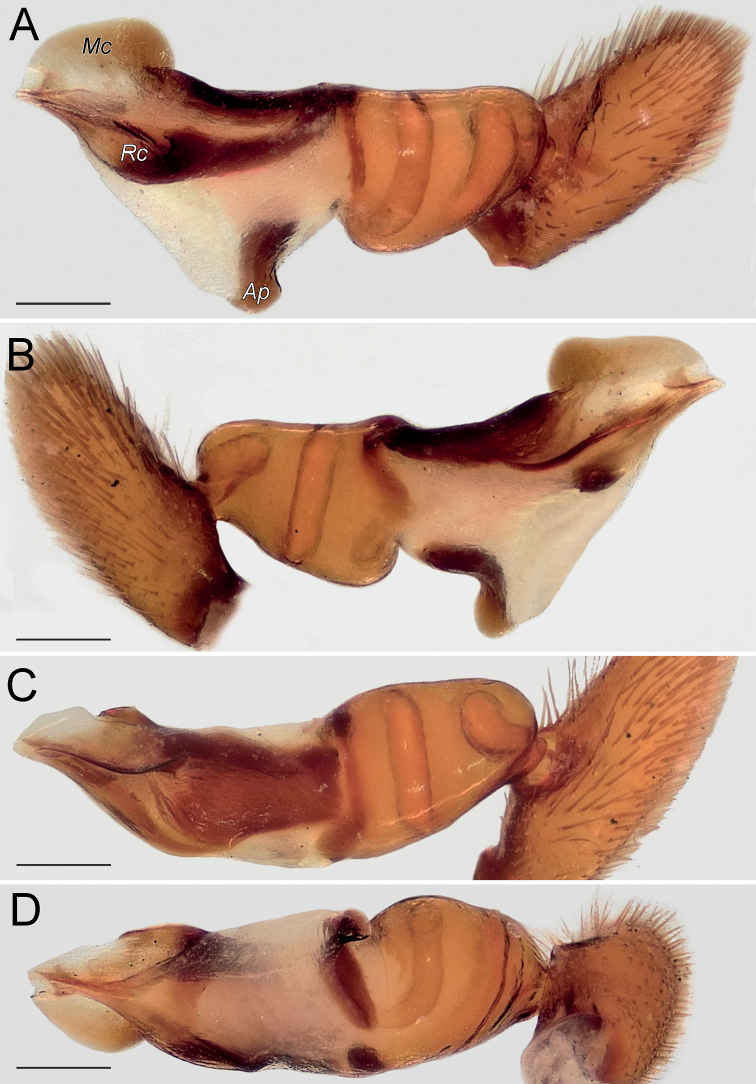
Male of *Dysderabakhtiari* sp. nov., bulb **A** retrolateral view **B** prolateral view **C** anterior view **D** posterior view. Scale bars: 0.25 mm. Abbreviations: *Ap* – posterior apophysis, *Mc* – median crest, *Rc* – retrolateral crest.

Palp as in Fig. 4A–D; bulb ca. 2× longer than wide; tegulum bell-shaped, 1.2× longer than wide; psembolus 1.5× longer than tegulum; median crest (*Mc*) rounded, ca. 2.2× shorter than length of psembolus, ca. 4× wider than high; posterior apophysis (*Ap*) very large, rounded; incision between tegulum and psembolus absent; retrolateral crest (*Rc*) gradually rounded.

**Female.** Unknown.

#### Distribution.

Known only from the type locality in Chaharmahal & Bakhtiari Province, southwestern Iran (Fig. 35).

### ﻿ Dysdera hormuzensis sp. nov.

Taxon classificationAnimaliaAraneaeDysderidae

245D23FC-CEC8-5241-BC6C-79DB4629F9C8

https://zoobank.org/0AAED40A-FE9B-4302-A6FB-71C20654D745

Figs 5A–C , 6A, B

#### Type material.

***Holotype*** ♀ (ZMUT), Iran: Hormozgan Province: Hormuz Island, 27°02'N, 56°29'E, 01.2014 (A. Zamani).

#### Etymology.

The specific epithet is an adjective referring to Hormuz Island, from where the holotype was collected.

#### Diagnosis.

The new species differs from all *Dysdera* species occurring in the region by the receptacle divided into two chambers (vs. undivided), and the indistinct dorsal arch (vs. distinct).

#### Description.

**Figure 5.**
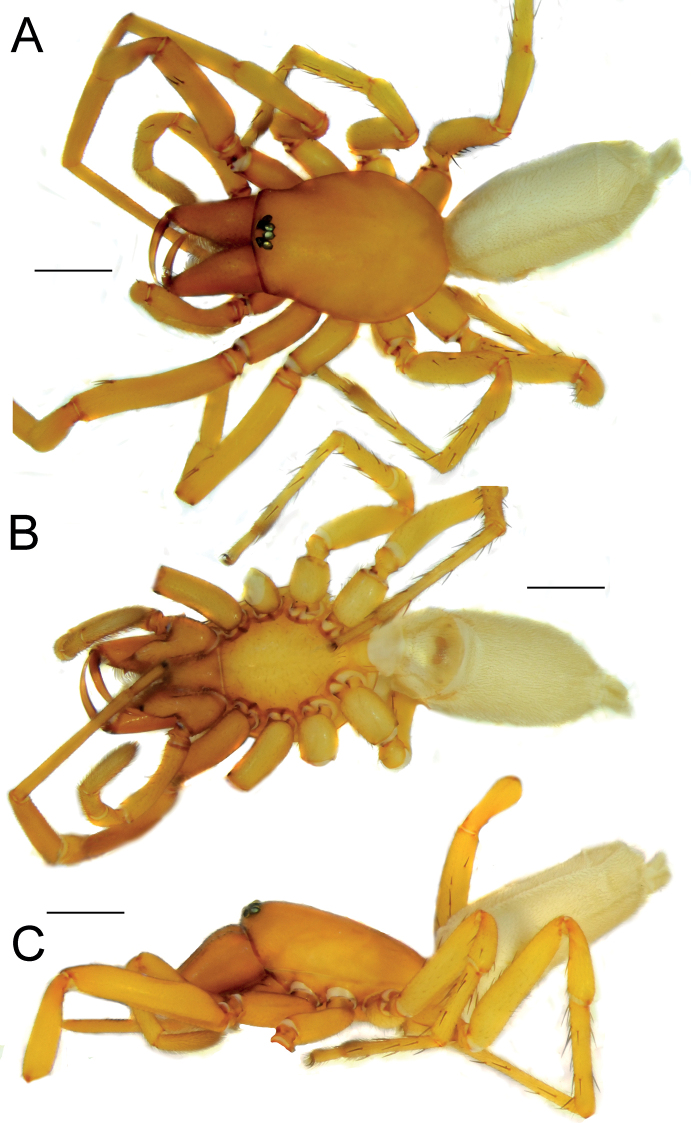
Female of *Dysderahormuzensis* sp. nov., habitus **A** dorsal view **B** ventral view **C** lateral view. Scale bars: 1.0 mm.

**Female.** Habitus as in Fig. 5A–C. Total length 8.11. Carapace 3.26 long, 2.56 wide. Eye diameters: AME 0.13, PME 0.13, PLE 0.14. Carapace, sternum, chelicerae, labium, and maxillae orangish. Legs pale orange. Abdomen pale cream-coloured, without any pattern. Spinnerets uniformly pale cream-coloured. Measurements of legs: I: 7.87 (2.19, 1.29, 2.01, 1.85, 0.53), II: 7.31 (1.99, 1.19, 1.77, 1.83, 0.53), III: 5.83 (1.61, 0.88, 1.13, 1.74, 0.47), IV: 7.27 (1.83, 1.05, 1.56, 2.22, 0.61). Spination: I: Fe: 2pl. II: Fe: 3pl. III: Fe: 3d; Pa: 1d, 1pl; Ti: 5pl, 2rl, 6v; Mt: 3d, 6pl, 2rl. IV: Fe: 8d; Pa: 1pl; Ti: 5pl, 3rl, 6v; Mt: 3pl, 4rl, 3v.

**Figure 6.**
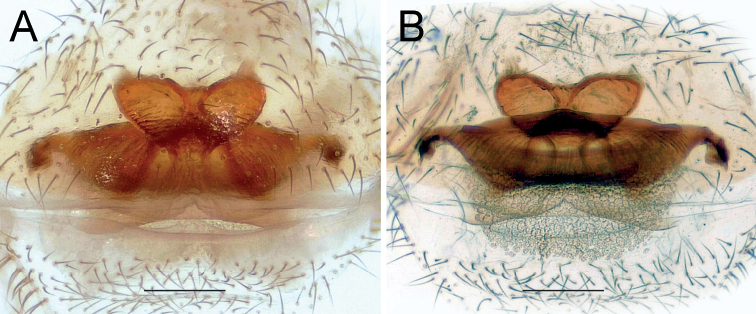
Female of *Dysderahormuzensis* sp. nov., endogyne **A** ventral view **B** dorsal view. Scale bars: 0.25 mm.

Endogyne as in Fig. 6A, B; length/width ratio ca. 2.6; receptacle 2× longer than wide, divided in two chambers, with an anterior median concavity; anterior angles located almost at mid-part of each chamber and directed anteriorly, approximately as long as wide; dorsal arch indistinct; transverse bar ca. 4.6× longer than wide, mid-part similar to an inverted trapezoid, anterior part 1.3× longer than posterior part; lateral edges directed postero-laterally, clearly separated from transverse bar by an incision; posterior diverticulum elongated horizontally.

**Male.** Unknown.

#### Comments.

The species group (or even generic) assignment of this species is tentative pending the collection of the corresponding male.

#### Distribution.

Known only from the type locality in Hormuz Island, the Persian Gulf (Fig. 35).

### ﻿ Dysdera iranica sp. nov.

Taxon classificationAnimaliaAraneaeDysderidae

D1F011A4-43AA-549C-9665-8F977D9D0D91

https://zoobank.org/DB4E6E44-A312-424C-9AA5-BBE9E6A06FB5

**Figure 7.**
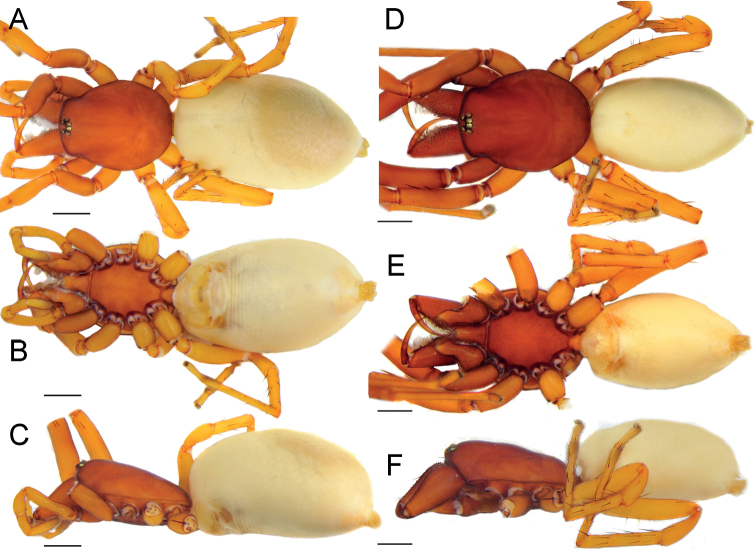
Female (**A–C**) and male (**D–F**) of *Dysderairanica* sp. nov., habitus **A, D** dorsal view **B, E** ventral view **C, F** lateral view. Scale bars: 1.0 mm.

**Figure 8.**
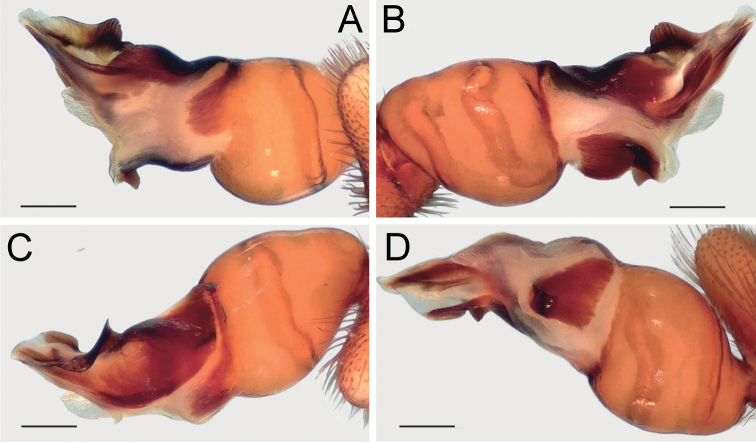
Male of *Dysderairanica* sp. nov., bulb **A** retrolateral view **B** prolateral view **C** anterior view **D** posterior view. Scale bars: 0.25 mm.

**Figure 9.**
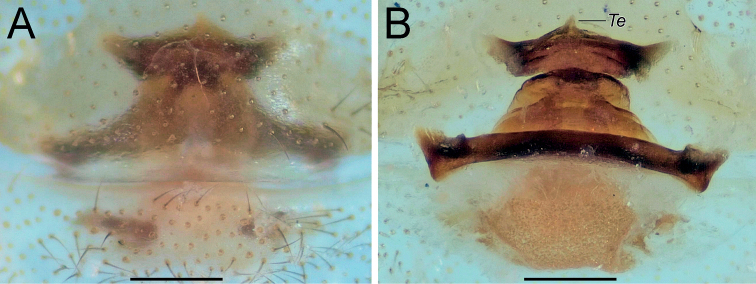
Female of *Dysderairanica* sp. nov., endogyne **A** ventral view **B** dorsal view. Scale bars: 0.25 mm. Abbreviation: *Te* – triangular extension.

Figs 7A–F , 8A–D , 9A, B

#### Type material.

***Holotype*** ♂ (ZMUT), Iran: Hormozgan Province: Siahu, 27°45'N, 56°20'E, 02.2018 (A. Zamani). ***Paratypes***: 1♀ (ZMUT), same data as the holotype; 1♂ (MMUE), Fars Province: Shiraz, inside house, 29°37'N, 52°30'E, 6.05.1982 (P. Hassanzadeh); 1♂ (MMUE), Shiraz, garden, 29°37'N, 52°30'E, 04.1982 (P. Hassanzadeh); 1♂1♀ (MMUE), Shiraz, inside house, 29°37'N, 52°30'E, 06.1982 (P. Hassanzadeh); 1♀ (ZMUT), Hormozgan Province: Siahu, 27°45'N, 56°20'E, 02.2020 (A. Zamani); 1♀ (ZMUT), Siahu, palm orchards, 27°45'N, 56°20'E, 02.2020 (A. Zamani).

#### Etymology.

The specific epithet is an adjective and refers to the country from where the specimens of the new species were collected.

#### Diagnosis.

The male of the new species is somewhat similar to that of *D.arabica* Deeleman-Reinhold, 1988 from Oman (cf. Fig. 8A–D and Deeleman-Reinhold and Deeleman 1988: figs 309–310), but differs by the small, claw-like posterior apophysis (vs. broad and rounded) and keel-like median crest (vs. rounded). The male of *D.iranica* sp. nov. differs from those of its congeners occurring in Iran by the elongate keel-like median crest (vs. rounded or triangular). The female of *D.iranica* sp. nov. is most similar to that of *D.tapuria* sp. nov. by having a very wide receptacle (i.e., > 2× wider than the transverse bar), but differs by having a triangular extension (*Te*) in the anterior margin of the receptacle (vs. absent).

#### Description.

**Male (Holotype).** Habitus as in Fig. 7D–F. Total length 7.32. Carapace 3.35 long, 2.63 wide. Eye diameters: AME 0.15, PME 0.15, PLE 0.15. Carapace, sternum, chelicerae, labium, and maxillae reddish. Legs yellowish orange. Abdomen cream-coloured, without any pattern. Spinnerets uniformly dark yellowish. Measurements of legs: I: 11.4 (3.14, 1.92, 2.86, 2.84, 0.63), II: 10.26 (2.72, 1.78, 2.46, 2.68, 0.62), III: 7.61 (2.14, 1.17, 1.54, 2.13, 0.63), IV: 9.75 (2.69, 1.53, 2.12, 2.74, 0.67). Spination: I: Fe: 3pl. II: Fe: 3pl. III: Fe: 3pl, 1rl; Pa: 1pl; Ti: 5pl, 3rl, 5v; Mt: 6pl, 2rl, 2v. IV: Fe: 7d, 2pl, 1rl; Pa: 1pl; Ti: 6pl, 2rl, 6v; Mt: 3pl, 2rl, 5v.

Palp as in Fig. 8A–D; bulb ca. 2.5× longer than wide; tegulum bell-shaped, almost as long as wide; psembolus 1.4× longer than tegulum; median crest rounded, ca. 2.5× shorter than length of psembolus, ca. 2.5× wider than high; posterior apophysis claw-shaped, 1.5× longer than wide; incision between tegulum and psembolus absent; retrolateral crest gradually rounded.

**Female.** Habitus as in Fig. 7A–C. Total length 8.20. Carapace 3.03 long, 2.45 wide. Eye diameters: AME 0.14, PME 0.13, PLE 0.14. Colouration as in male. Measurements of legs: I: 8.45 (2.48, 1.44, 1.94, 2.01, 0.58), II: 7.79 (2.17, 1.35, 1.88, 1.91, 0.48), III: 6.25 (1.77, 1.05, 1.16, 1.71, 0.56), IV: 7.88 (2.28, 1.29, 1.72, 2.01, 0.58). Spination: I: Fe: 3pl. II: Fe: 2pl. III: Fe: 2d, 3pl, 3rl; Ti: 4pl, 2rl, 5v; Mt: 5pl, 2rl, 3v. IV: Fe: 7d, 1rl; Ti: 3pl, 2rl, 6v; Mt: 3pl, 2rl, 3v.

Endogyne as in Fig. 9A, B; length/width ratio ca. 2.1; receptacle 4× longer than wide, almost inverted trapezoidal; anterior angle large and triangular, with its base as wide as receptacle, directed antero-laterally; receptacle with median triangular extension (*Te*); dorsal arch trapezoidal, anterior corners rounded, posterior margin 1.6× longer than anterior, anterior margin ca. 1.25× longer than width of dorsal arch; transverse bar slightly arched, 1.5× longer than receptacle; lateral edges with horizontal anterior margins; posterior diverticulum trapezoidal.

#### Distribution.

Known from the listed localities in Fars and Hormozgan provinces, south-central and southern Iran (Fig. 35).

### ﻿ Dysdera isfahanica sp. nov.

Taxon classificationAnimaliaAraneaeDysderidae

CA37509D-B3BD-5DE4-B503-426AA9B08763

https://zoobank.org/BDD33AFB-357C-4213-8EC6-2D30E261FE4B

**Figure 10.**
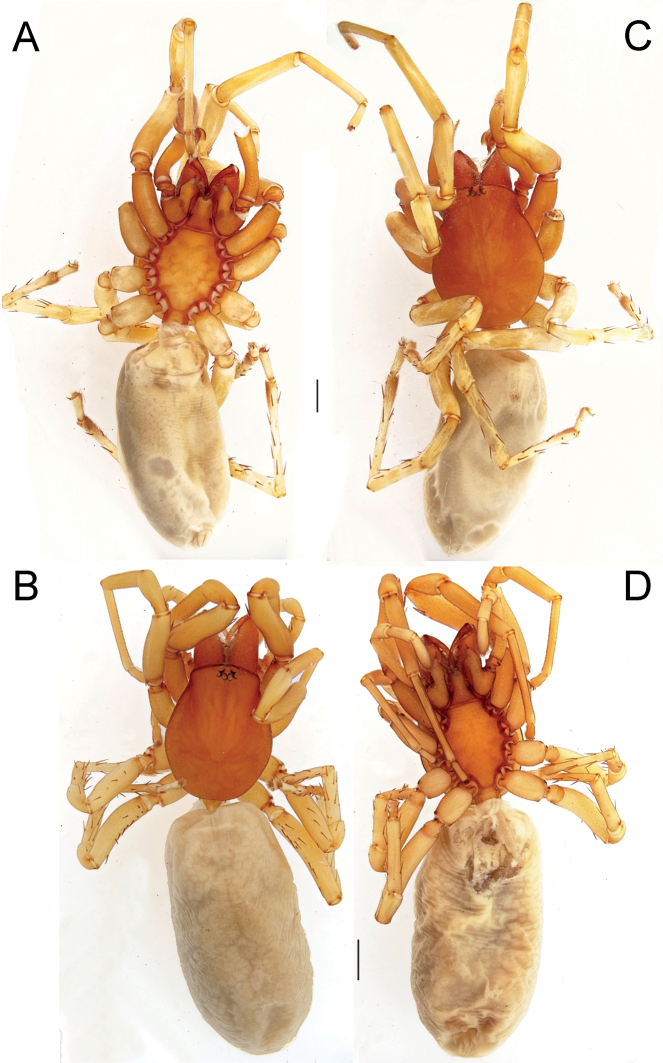
Male (**A, C**) and female (**B, D**) of *Dysderaisfahanica* sp. nov., habitus **A, D** ventral view **B, C** dorsal view. Scale bars: 1.0 mm.

**Figure 11.**
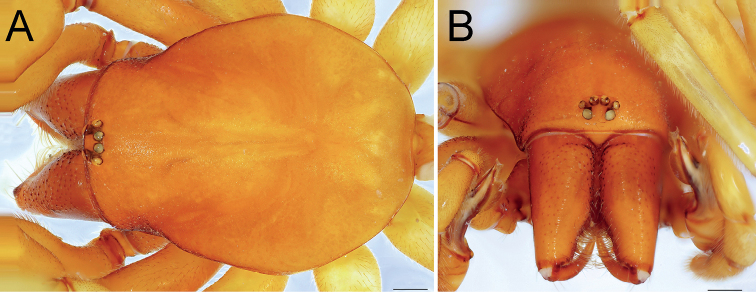
Male of *Dysderaisfahanica* sp. nov., prosoma **A** dorsal view **B** frontal view. Scale bars: 0.5 mm.

**Figure 12.**
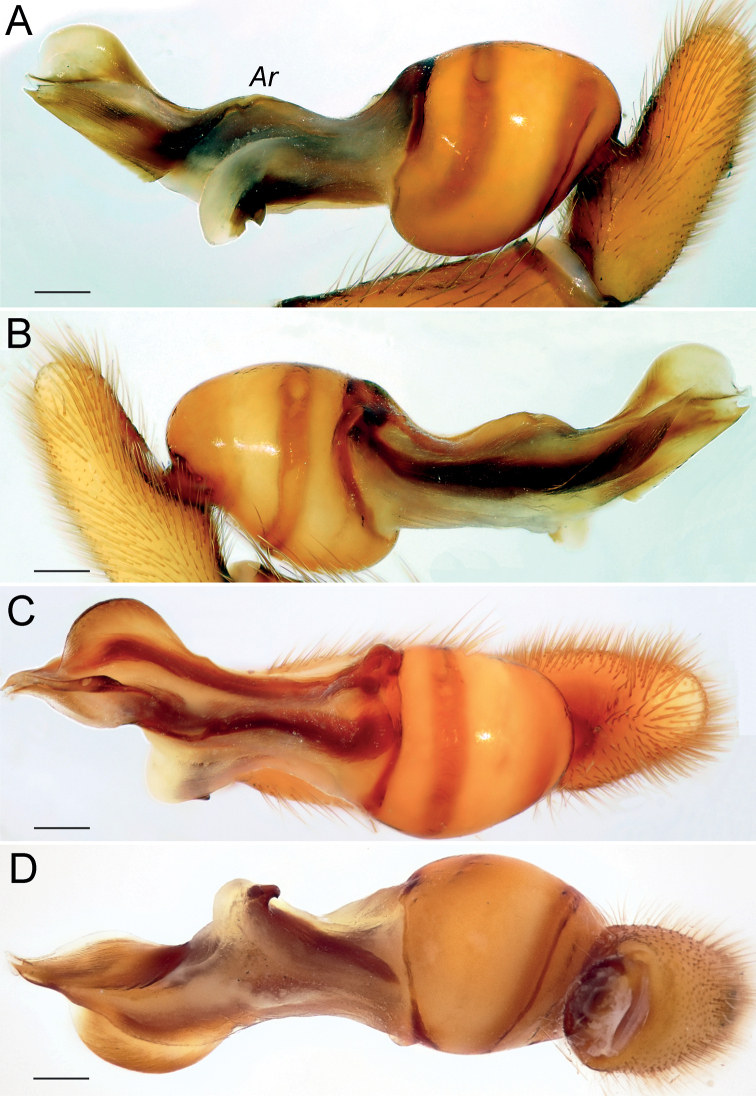
Male of *Dysderaisfahanica* sp. nov., bulb **A** retrolateral view **B** prolateral view **C** anterior view **D** posterior view. Scale bars: 0.25 mm. Abbreviation: *Ar* – arch-like ridge.

Figs 10A–D , 11A, B , 12A–D , 14A–D

Dysdera erythrina : Roewer 1955: 752 (in part, misidentification).

#### Type material.

***Holotype*** ♂ (SMF), Iran: Isfahan Province: Pir Bakran, 150 km west of Isfahan, 32°28'N, 51°33'E (H. Löffler). ***Paratype***: 1♀ (SMF), same data as the holotype.

#### Etymology.

The specific epithet is an adjective, referring to the type locality of the species.

#### Diagnosis.

The male of this species differs from those of the other species of the *aculeata* group occurring in the region by the very long psembolus (i.e., length of psembolus/length of tegulum = 1.85 in the new species, vs. 1.6 or less in most other species), rounded arch-like ridge (*Ar*), presence of the notch of posterior apophysis (vs. absent), and median position of the posterior apophysis on the psembolus (vs. close to tegulum). *Dysderapersica* sp. nov. also bears a long psembolus (i.e., length of psembolus/length of tegulum = 2), but differs from *D.isfahanica* sp. nov. in the shape of the posterior apophysis. The female of this species can be recognized by its long anterolaterally stretched angles of the receptacle.

#### Description.

**Male.** Habitus as in Figs 10A, C, 11A, B. Total length 10.47. Carapace 4.15 long, 3.45 wide. Eye diameters: AME 0.17, PME 0.14, PLE 0.15. Carapace, sternum, chelicerae, labium, and maxillae orange. Legs yellowish. Abdomen pale beige, without any pattern. Spinnerets uniformly pale beige. Measurements of legs: I: 13.57 (3.77, 2.47, 3.23, 3.21, 0.89), II: 12.83 (3.47, 2.34, 2.96, 3.16, 0.90), III: 9.23 (2.60, 1.39, 1.95, 2.58, 0.71), IV: 12.28 (3.47, 1.91, 2.59, 3.43, 0.88). Spination: I: Fe: 1–2pl. II: no spines. III: Ti: 4pl, 2rl, 4v; Mt: 6pl, 6rl. IV: Fe: 6d; Ti: 2d, 2pl, 2rl, 2v; Mt: 6pl, 5rl.

Palp as in Fig. 12A–D; bulb ca. 2.5× longer than wide; tegulum bell-shaped, almost as long as wide; psembolus 1.8× longer than tegulum; median crest rounded, ca. 3.4× shorter than length of psembolus, ca. 2× wider than high; posterior apophysis claw-like, 2.3× longer than wide; incision between tegulum and psembolus absent; retrolateral crest forming right angle.

**Female.** Habitus as in Fig. 10B, D. Total length 9.45. Carapace 3.30 long, 2.55 wide. Eye diameters: AME 0.15, PME 0.14, PLE 0.12. Colouration as in male. Measurements of legs: I: 9.74 (2.76, 1.66, 2.31, 2.40, 0.61), II: 8.80 (2.52, 1.50, 2.08, 2.13, 0.57), III: 6.74 (1.94, 1.08, 1.28, 1.90, 0.54), IV: 8.68 (2.47, 1.31, 1.90, 2.46, 0.54). Spination: I: Fe: 3pl. II: Fe: 4pl. III: Fe: 5d, 1rl; Pa: 3pl; Ti: 4pl, 2rl, 6v; Mt: 3pl, 2rl, 6v. IV: Fe: 8–11d, 2pl; Pa: 1pl; Ti: 5pl, 4rl, 6v; Mt: 2pl, 3rl, 6v.

Endogyne as in Fig. 14A–D; length/width ratio ca. 2; receptacle ca. 5× longer than wide, with long anterior angles (i.e., longer than width of their bases), anterior margin almost straight; dorsal arch trapezoidal; transverse bar straight, 1.6× longer than receptacle; anterior margin of lateral edge inclined, lateral edge approximately as wide as receptacle; posterior diverticulum almost rectangular, its posterior edge rounded.

#### Comments.

The material of this species and *D.mazeruni* sp. nov. (i.e., one male and two females in total) were reported by Roewer (1955); although he indicated that the females were collected in two different localities, they were found preserved in the same vial. The paratype female of *D.isfahanica* sp. nov. is matched with the holotype male due to their similar spination pattern and colouration.

#### Distribution.

Known only from the type locality in Isfahan Province, central Iran (Fig. 35).

### ﻿ Dysdera mazeruni sp. nov.

Taxon classificationAnimaliaAraneaeDysderidae

83EF3B31-E0E7-5DF7-94A2-906ED9D5CE02

https://zoobank.org/53699EB1-066B-405B-872C-84E69085B8F6

Figs 13A, B , 14E–G

Dysdera erythrina : Roewer 1955: 752 (in part, misidentification).

#### Type material.

***Holotype*** ♀ (SMF), Iran: Mazandaran Province: Caspian coast, forest in Chalus, 36°40'N, 51°25'E (F. Starmühlner). ***Paratype***: 1♀ (MMUE), north of Javaher-Deh Vil., ~ 500 m down by elevation down from vil., 36°52'19.2"N, 50°28'01.2"E, 9.06.2000 (Y.M. Marusik).

#### Etymology.

The specific epithet is a noun in apposition, named after an Iranian language of the northwestern branch spoken by the Mazandarani people.

#### Diagnosis.

The new species is similar to *D.isfahanica* sp. nov., but differs by the arched anterior margin of receptacle (vs. almost straight), almost square-shaped dorsal arch (vs. distinctly trapezoidal), and shorter anterior angles (vs. longer, cf. Fig. 14A–D, E–G).

#### Description.

**Figure 13.**
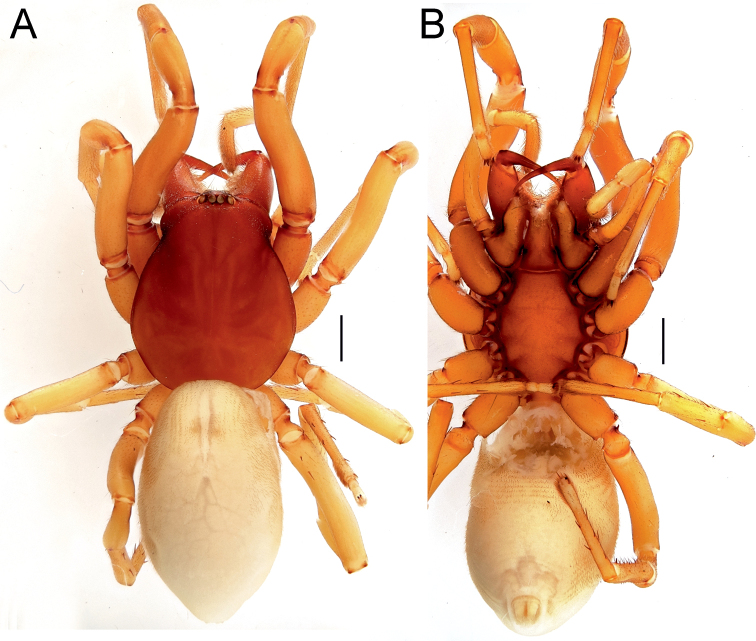
Female of *Dysderamazeruni* sp. nov., habitus **A** dorsal view **B** ventral view. Scale bars: 1.0 mm.

**Female (Holotype).** Habitus as in Fig. 13A, B. Total length 8.90. Carapace 4.25 long, 3.37 wide. Eye diameters: AME 0.21, PME 0.19, PLE 0.22. Carapace, sternum, chelicerae, labium, and maxillae orange. Legs yellowish. Abdomen pale beige, without any pattern. Spinnerets uniformly pale beige. Measurements of legs: I: 10.93 (3.48, 2.02, 2.50, 2.25, 0.68), II: 10.00 (2.95, 1.86, 2.31, 2.25, 0.63), III: 7.46 (2.38, 1.08, 1.44, 1.91, 0.65), IV: 9.92 (2.71, 1.50, 2.12, 2.88, 0.71). Spination: I, II: no spines. III: Fe: 1d; Ti: 2pl, 2rl, numerous v spine-like setae; Mt: 2pl, 4rl, 5v. IV: Fe: 2d; Ti: 2pl, 2rl, numerous v spine-like setae; Mt: 3pl, 2rl, 6v.

**Figure 14.**
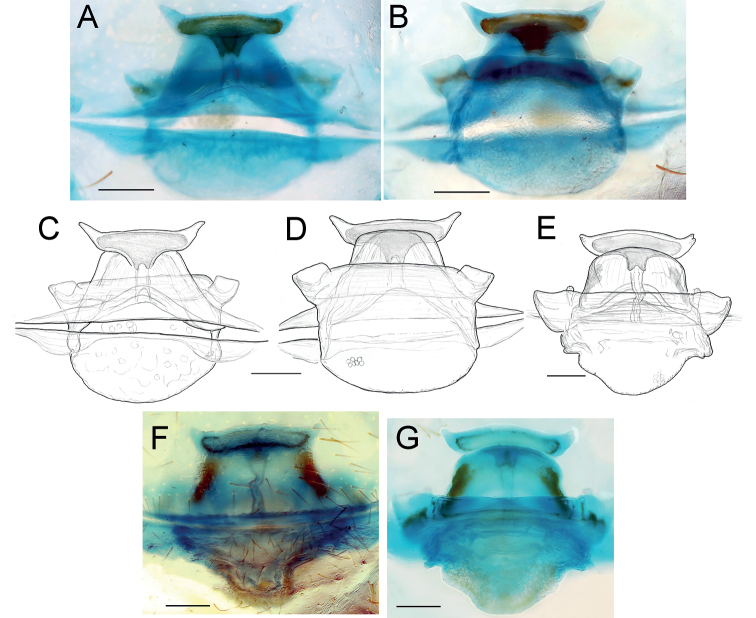
Females of *Dysderaisfahanica* sp. nov. (**A–D**) and *D.mazeruni* sp. nov. (**E–G**), endogynes **A, C, F** ventral view **B, D, E, G** dorsal view. Scale bars: 0.2 mm.

Endogyne as in Fig. 14E–G; length/width ratio ca. 2.2; receptacle with slightly arched anterior margin, ca. 5× longer than wide, anterior angles slightly rounded; dorsal arch almost square-shaped, posterior margin 1.2× longer than anterior margin; transverse bar straight, 1.7× longer than receptacle; lateral edges broad, wider than receptacle; posterior diverticulum narrowing posteriorly.

**Male.** Unknown.

#### Comments.

As for the previous species.

#### Distribution.

Known only from the listed localities in Mazandaran Province, northern Iran (Fig. 35).

### ﻿ Dysdera persica sp. nov.

Taxon classificationAnimaliaAraneaeDysderidae

4E0DFB13-0C1E-5401-9D88-770C09E499B6

https://zoobank.org/5CCD54FC-78A6-4AA9-A521-C53C8C70F140

**Figure 15.**
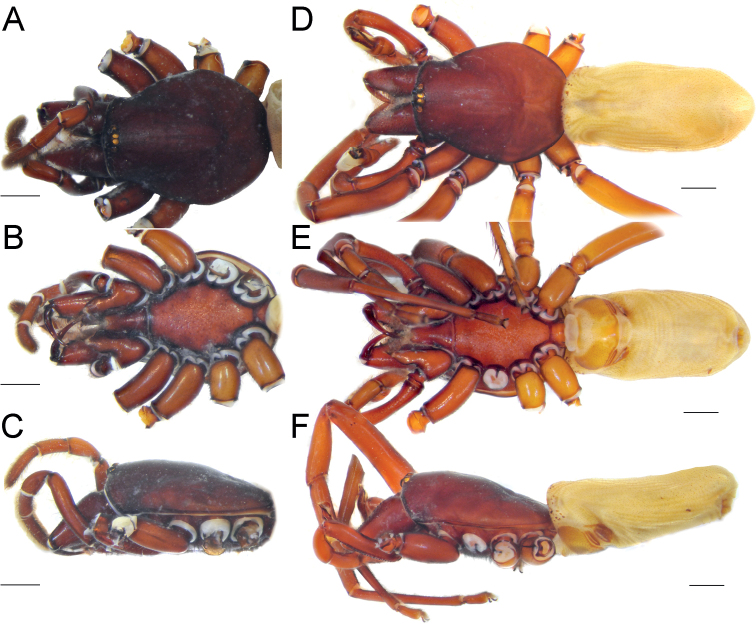
Female (**A–C**) and male (**D–F**) of *Dysderapersica* sp. nov., habitus **A, D** dorsal view **B, E** ventral view **C, F** lateral view. Scale bars: 1.0 mm.

**Figure 16.**
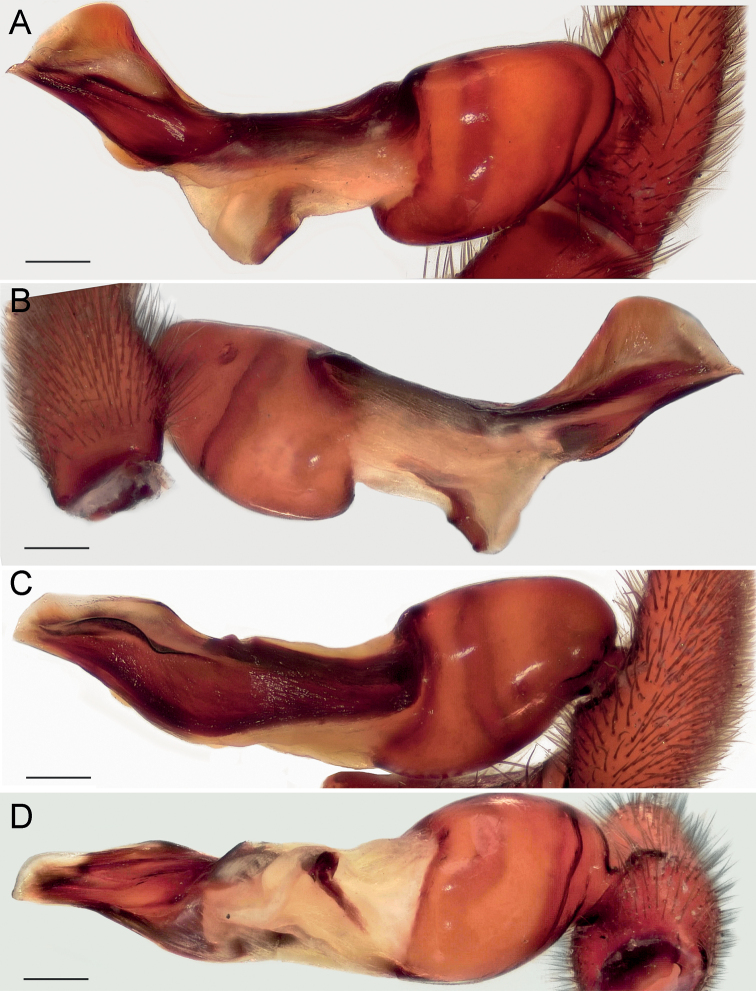
Male of *Dysderapersica* sp. nov., bulb **A** retrolateral view **B** prolateral view **C** anterior view **D** posterior view. Scale bars: 0.25 mm.

**Figure 17.**
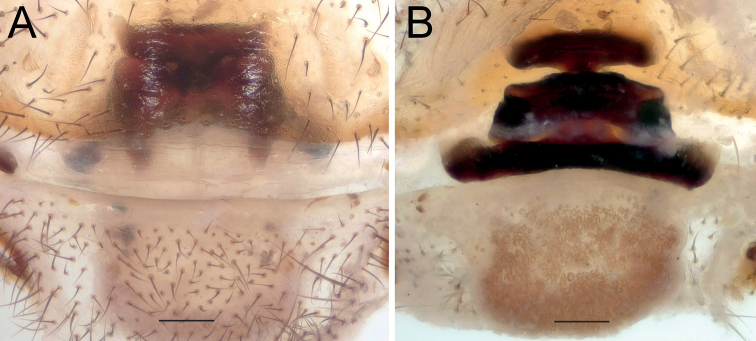
Female of *Dysderapersica* sp. nov., endogyne **A** ventral view **B** dorsal view. Scale bars: 0.25 mm.

Figs 15A–F , 16A–D , 17A, B

#### Type material.

***Holotype*** ♂ (ZMUT), Iran: Golestan Province: Shast Kalateh, 36°45'N, 54°21'E, 2017 (R. Rafiei-Jahed). ***Paratypes***: 7♂5♀ (ZMUT), same data as the holotype; 1♂ (ZMUT), Mazandaran Province: Polur, 35°50'N, 52°03'E, 10.2015 (A. Zamani); 1♂ (MHNG), Kiyasar, 36°14'N, 53°33'E, 1500 m, 11.07.1975 (A. Senglet).

#### Etymology.

The specific epithet is an adjective, referring to the historical region of the Middle East, located in eastern Mesopotamia, which is now Iran.

#### Diagnosis.

The male of this species differs from those of the other species of the *aculeata* group occurring in Iran by the extremely long bulb (especially psembolus, i.e., twice longer than tegulum), and by the tegulum with posterior margin 1.3× longer than anterior margin (vs. equal or shorter in length). The female of *D.persica* sp. nov. differs from those of its congeners by having the broadest dorsal arch, bearing almost angled anterior corners (vs. rounded).

#### Description.

**Male (Holotype).** Habitus as in Fig. 15D–F. Total length 9.06. Carapace 4.19 long, 3.20 wide. Eye diameters: AME 0.14, PME 0.16, PLE 0.17. Carapace, sternum, chelicerae, labium, and maxillae reddish brown. Legs orange. Abdomen greyish, without any pattern. Spinnerets uniformly dark yellowish. Measurements of legs: I: 13.98 (3.96, 2.36, 3.43, 3.52, 0.71), II: 12.87 (3.60, 2.11, 3.07, 3.27, 0.82), III: 9.38 (2.74, 1.51, 1.84, 2.70, 0.59), IV: 12.37 (3.54, 1.95, 2.66, 3.40, 0.82). Spination: I: Fe: 2pl. II: Fe: 1pl. III: Fe: 1pl; Ti: 5pl, 3rl, 4v; Mt: 5d, 9pl, 5rl, 3v. IV: Fe: 4d; Ti: 4pl, 6rl, 1v; Mt: 5d, 6pl, 7rl, 6v. Ti III–IV with a row of thin rigid ventral setae.

Palp as in Fig. 16A–D; bulb ca. 2.3× longer than wide; tegulum bell-shaped, almost as long as wide; psembolus 2× longer than tegulum; median crest triangular, ca. 2× shorter than length of psembolus, ca. 2.4× wider than high; posterior apophysis broad; incision between tegulum and psembolus present; retrolateral crest gradually rounded.

**Female.** Habitus as in Fig. 15A–C. Total length 11.0. Carapace 4.25 long, 3.16 wide. Eye diameters: AME 0.17, PME 0.18, PLE 0.15. Carapace and chelicerae dark reddish violet; sternum, labium, and maxillae reddish brown. Leg reddish brown. Abdomen greyish, without any pattern. Spinnerets uniformly greyish. Measurements of legs: I: 11.02 (3.15, 1.98, 2.59, 2.51, 0.79), II: 9.61 (2.72, 1.76, 2.32, 2.14, 0.67), III: 8.07 (2.27, 1.33, 1.62, 2.14, 0.71), IV: 10.54 (3.02, 1.66, 2.15, 2.89, 0.82). Spination: I: Fe: 3pl. II: Fe: 1pl. III: Fe: 1d; Ti: 4pl, 4rl; Mt: 12pl, 5rl, 4v. IV: Fe: 4d; Ti: 5pl, 4rl, 1v; Mt: 14pl, 9rl, 3v. Ti III–IV with a row of thin rigid ventral setae.

Endogyne as in Fig. 17A, B; length/width ratio ca. 1.9; receptacle 3.5× longer than wide, 1.2× wider than transverse bar; anterior angles rounded; dorsal arch trapezoidal; transverse bar straight, ca. 1.8× longer than receptacle; lateral edges with almost horizontal anterior margins, as long as width of receptacle; posterior diverticulum rectangular.

#### Distribution.

Known only from listed localities in Golestan and Mazandaran provinces, northern Iran (Fig. 35).

### ﻿ Dysdera pococki

Taxon classificationAnimaliaAraneaeDysderidae

Dunin, 1985

64C95D09-4759-543D-97BA-74A53233F151

Dysdera concinna : Pocock 1889: 112 (misidentified as per Deeleman-Reinhold and Deeleman 1988: 236).
Dysdera pococki Dunin 1985: 114, figs 1, 2 (♂).

#### Comments.

The Iranian record of this species is doubtful. Dunin (1985) described *D.pococki* on the basis of a male specimen from Turkmenistan, and the female of the species remains undescribed. Without providing any illustrations, Pocock (1889) reported a single female specimen from northeastern Iran which he tentatively identified as *D.concinna* L. Koch, 1878; this record was later attributed to *D.pococki* by Deeleman-Reinhold and Deeleman (1988), due to their close collection localities and without an examination of the Iranian material. This matter should be revisited once the female of *D.pococki* is described and the specimen reported by Pocock from Iran is studied and illustrated.

#### Records in Iran.

Razavi Khorasan (Pocock 1889) (Fig. 35).

#### Distribution.

Iran, Turkmenistan.

### ﻿ Dysdera sagartia sp. nov.

Taxon classificationAnimaliaAraneaeDysderidae

9671F65B-F763-5D5D-BC74-4D187D1FB975

https://zoobank.org/FD19AF41-B7A5-4B99-A384-9FEB0D8B3E77

**Figure 18.**
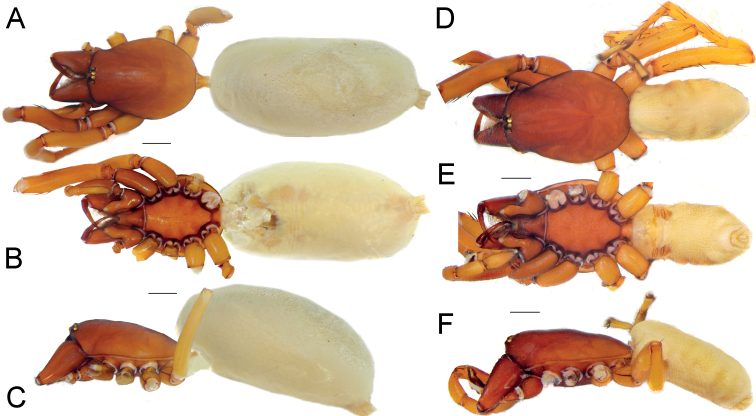
Female (**A–C**) and male (**D–F**) of *Dysderasagartia* sp. nov., habitus **A, D** dorsal view **B, E** ventral view **C, F** lateral view. Scale bars: 1.0 mm.

**Figure 19.**
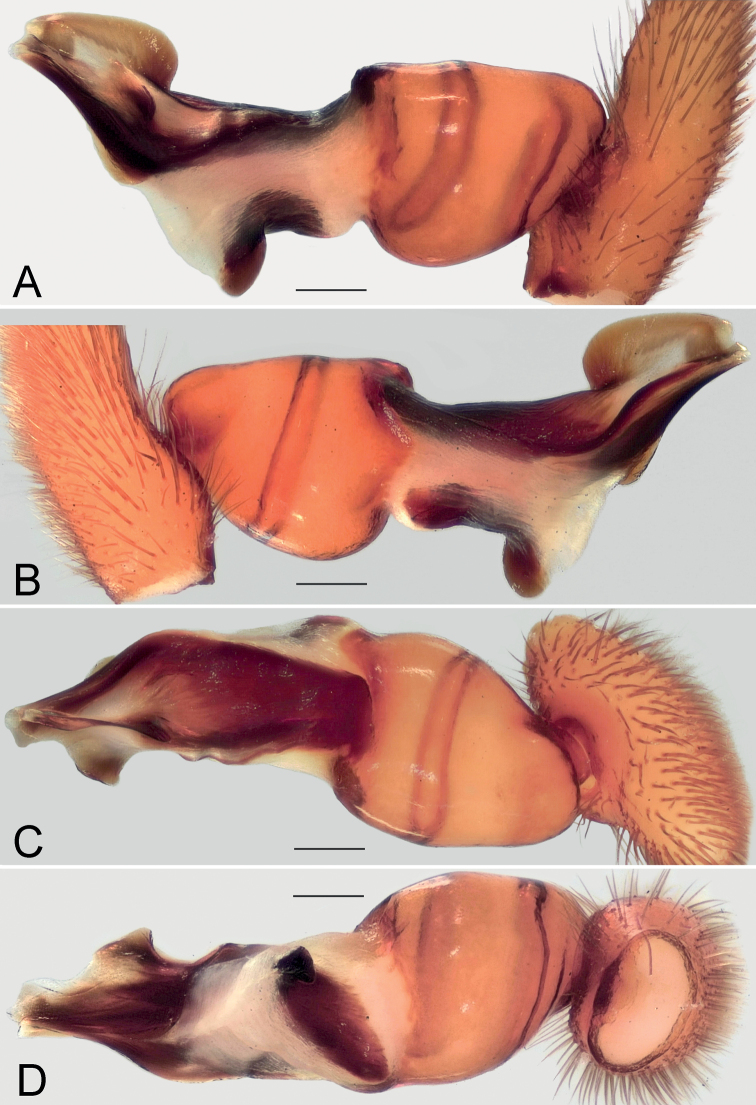
Male of *Dysderasagartia* sp. nov., bulb **A** retrolateral view **B** prolateral view **C** anterior view **D** posterior view. Scale bars: 0.25 mm.

**Figure 20.**
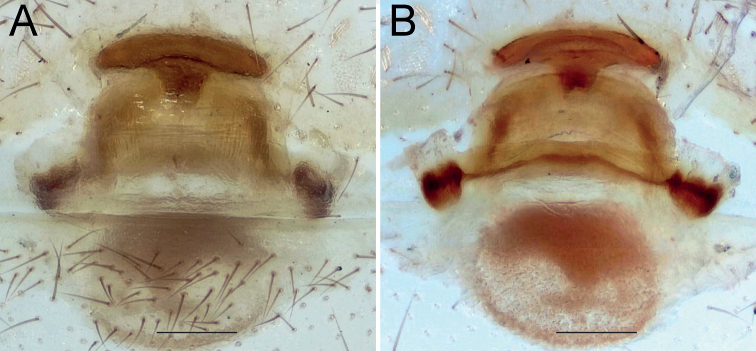
Female of *Dysderasagartia* sp. nov., endogyne **A** ventral view **B** dorsal view. Scale bars: 0.25 mm.

Figs 18A–F , 19A–D , 20A, B

#### Type material.

***Holotype*** ♂ (ZMUT), Iran: Tehran Province: Tehran, 35°45'N, 51°24'E, 04.2014 (A. Zamani). ***Paratypes***: 1♂1♀ (ZMUT), Tehran, 35°42'N, 51°25'E, 03.2014 (A.H. Bakhtiari).

#### Etymology.

The specific epithet is a noun in apposition, referring to an ancient tribe dwelling in the Iranian plateau.

#### Diagnosis.

The male of this species differs from those of the other species of the *aculeata* group occurring in Iran by the strong dorsal incision between tegulum and psembolus, and the posterior apophysis bent on right angle (vs. no or small incision, and posterior apophysis not bent on right angle); the most similar species is *D.mikhailovi* Fomichev & Marusik, 2021 from Tajikistan, from which the new species differs by having a dorsal incision between tegulum and psembolus, the parallel dorsal sides of tegulum and psembolus (vs. dorsal margin of psembolus inclined), and smaller retrolateral crest angled at distal 1/3 of psembolus (vs. larger and angled at mid-part). The female of *D.sagartia* sp. nov. differs from those of its congeners occurring in Iran by having an arched anterior margin of receptacle in combination with a lack of anterior angles (vs. species with arched receptacle have anterior angles), and the almost semiround dorsal arch (vs. trapezoidal).

#### Description.

**Male (Holotype).** Habitus as in Fig. 18D–F. Total length 8.38. Carapace 4.29 long, 3.17 wide. Eye diameters: AME 0.18, PME 0.15, PLE 0.15. Carapace, sternum, chelicerae, labium, and maxillae reddish. Legs yellowish orange. Abdomen cream-coloured, without any pattern. Spinnerets uniformly cream-coloured. Measurements of legs: I: 14.51 (3.99, 2.46, 3.61, 3.68, 0.77), II: 12.87 (3.41, 2.02, 3.19, 3.50, 0.75), III: 9.24 (2.75, 1.17, 2.04, 2.47, 0.81), IV: 11.84 (3.51, 1.62, 2.70, 3.13, 0.88). Spination: I: Fe: 3pl. II: Fe: 3pl. III: Fe: 1d, 3pl; Ti: 5pl, 2rl, 5v; Mt: 6pl, 4rl, 2v. IV: Fe: 8d, 1pl, 1rl; Ti: 6pl, 3rl, 7v; Mt: 6pl, 2rl, 5v.

Palp as in Fig. 19A–D; bulb ca. 2.1× longer than wide; tegulum bell-shaped, almost as long as wide; psembolus 1.44× longer than tegulum; median crest rounded, ca. 2.3× shorter than length of psembolus, ca. 3.1× wider than high; posterior apophysis broad; incision between tegulum and psembolus present; retrolateral crest bent on obtuse, almost right, angle.

**Female.** Habitus as in Fig. 18A–C. Total length 11.7. Carapace 3.85 long, 2.91 wide. Eye diameters: AME 0.16, PME 0.14, PLE 0.12. Colouration as in male. Measurements of legs: I: 10.60 (2.87, 1.75, 2.62, 2.73, 0.63), II: 11.38 (3.24, 1.86, 3.04, 2.81, 0.43), III: 8.27 (2.46, 1.27, 1.60, 2.32, 0.62), IV: 10.86 (3.07, 1.50, 2.36, 3.16, 0.77). Spination: I: Fe: 3pl. II: Fe: 3pl. III: Fe: 1d, 1pl; Ti: 6pl, 2rl, 5v; Mt: 2d, 4pl, 2rl, 3v. IV: Fe: 7d; Ti: 2pl, 2rl, 8v; Mt: 2d, 4pl, 5v.

Endogyne as in Fig. 20A, B; length/width ratio ca. 2; receptacle slightly arched, ca. 4.7× longer than wide, anterior angles indistinct; dorsal arch semi-oval, its posterior margin 1.5× longer than anterior; transverse bar 1.5× longer than receptacle and approximately as wide, slightly arched, lateral edges longer than wide; posterior diverticulum rounded.

#### Distribution.

Known only from the listed localities in Tehran Province, northern Iran (Fig. 35).

### ﻿ Dysdera verkana sp. nov.

Taxon classificationAnimaliaAraneaeDysderidae

AFD3A047-BC9C-5FB6-9377-ABA45FFAB955

https://zoobank.org/2E18F484-6E0E-4296-B253-285A0E98E268

Figs 21A–C , 22A–D

#### Type material.

***Holotype*** ♂ (ZMUT), Iran: Golestan Province: Azadshahr County, Khosh Yeylaq, 36°49'N, 55°20'E, 15.06.2016 (D. Kasatkin).

#### Etymology.

The specific epithet is a noun in apposition, referring to an Old Persian word for the Gorgan region, meaning “land of wolves”.

#### Diagnosis.

The male of the new species is most similar to that of *D.sagartia* sp. nov., but differs by the more rounded median crest, the posterior apophysis not bent on right angle (cf. Fig. 22A and Fig. 19A), and the relatively longer psembolus (i.e., length of psembolus/length of tegulum = 1.66 in *D.verkana* sp. nov., vs. 1.44 in *D.sagartia* sp. nov.).

#### Description.

**Figure 21.**
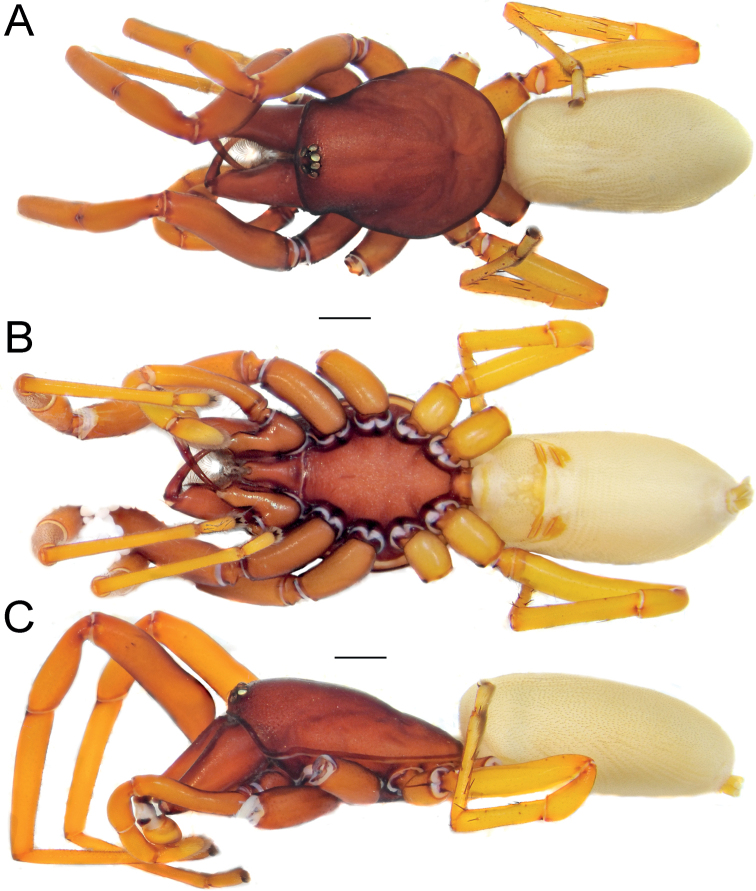
Male of *Dysderaverkana* sp. nov., habitus **A** dorsal view **B** ventral view **C** lateral view. Scale bars: 1.0 mm.

**Male.** Habitus as in Fig. 21A–C. Total length 10.8. Carapace 5.65 long, 4.26 wide. Eye diameters: AME 0.24, PME 0.23, PLE 0.21. Carapace, sternum, chelicerae, labium, and maxillae reddish brown. Legs orange. Abdomen cream-coloured, without any pattern. Spinnerets uniformly dark yellowish. Measurements of legs: I: 13.04 (3.90, 2.23, 3.10, 3.06, 0.75), II: 11.76 (3.39, 1.98, 2.91, 2.71, 0.77), III: 8.56 (2.64, 1.26, 1.69, 2.33, 0.64), IV: 11.46 (3.32, 1.76, 2.48, 3.20, 0.70). Spination: I: Fe: 2pl. II: Fe: 1pl. III: Fe: 1d, 2pl; Ti: 4pl, 2rl, 5v; Mt: 3pl, 2rl, 6v. IV: Fe: 6d, 1pl; Ti: 4pl, 4rl, 5v; Mt: 4pl, 2rl, 6v.

**Figure 22.**
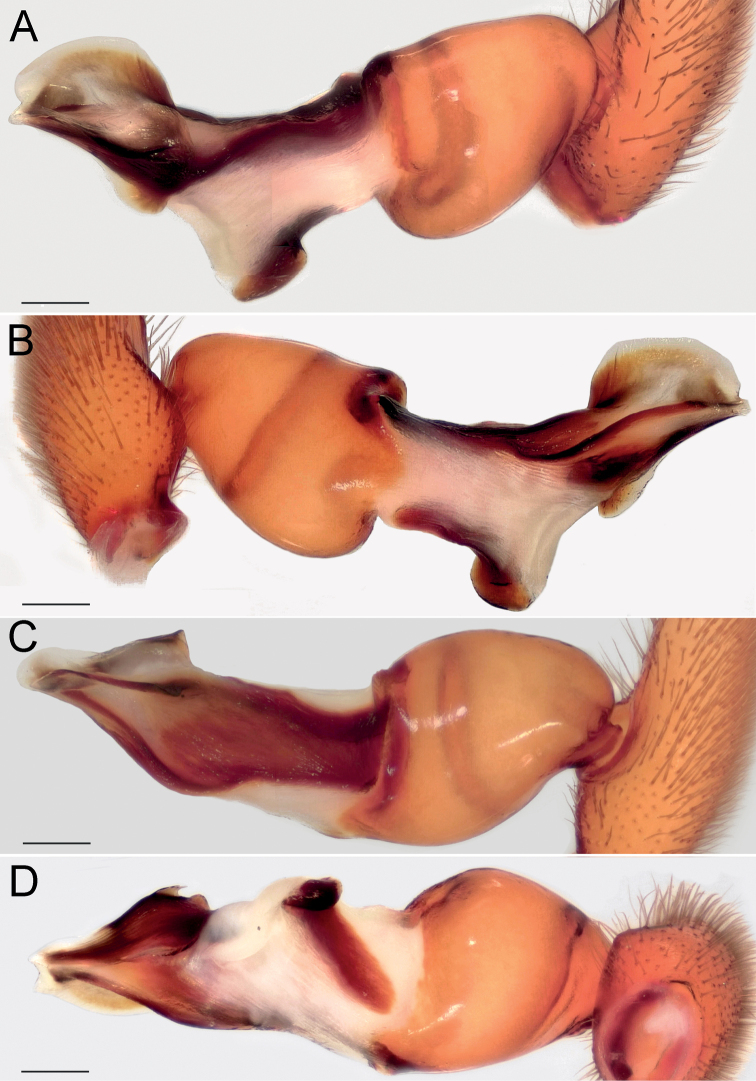
Male of *Dysderaverkana* sp. nov., bulb **A** retrolateral view **B** prolateral view **C** anterior view **D** posterior view. Scale bars: 0.25 mm.

Palp as in Fig. 22A–D; bulb ca. 2.2× longer than wide; tegulum bell-shaped, almost as long as wide; psembolus 1.66× longer than tegulum; median crest rounded, ca. 2.3× shorter than length of psembolus, ca. 2.7× wider than high; posterior apophysis broad; incision between tegulum and psembolus present; retrolateral crest roundly bent, forming right angle.

**Female.** Unknown.

#### Distribution.

Known only from the type locality in Golestan Province, northern Iran (Fig. 35).

##### ﻿*crocata* species group

**Diagnosis.** This group can be diagnosed by a combination of the following characters: the chelicerae straight or anteriorly convergent and longer than half of the length of the carapace, carapace broad and flat, and bulb with small or no lateral projection (Deeleman-Reinhold and Deeleman 1988).

### ﻿ Dysdera xerxesi sp. nov.

Taxon classificationAnimaliaAraneaeDysderidae

7ADCDF8A-AA3B-50F8-8C89-E45A9071A4D2

https://zoobank.org/84EDB4F0-007B-445B-A76D-C163B290ADFF

Figs 23A–C , 24A–D

#### Type material.

***Holotype*** ♂ (ZMUT), Iran: Bushehr Province: Asaluyeh, 27°20'N, 52°49'E, 27.01.2016 (A. Zamani).

#### Etymology.

The new species is named after Xerxes I, the fourth King of Kings of the Achaemenid Empire, ruling from 486 to 465 BC; adjective.

#### Diagnosis.

The new species differs from all of its congeners occurring in the region by having a stylus (*St*), rounded median crest (*Mc*) and wide posterior apophysis (*Ap*); none of the other species has a rounded median crest, and those with a stylus, have a small posterior apophysis.

#### Description.

**Figure 23.**
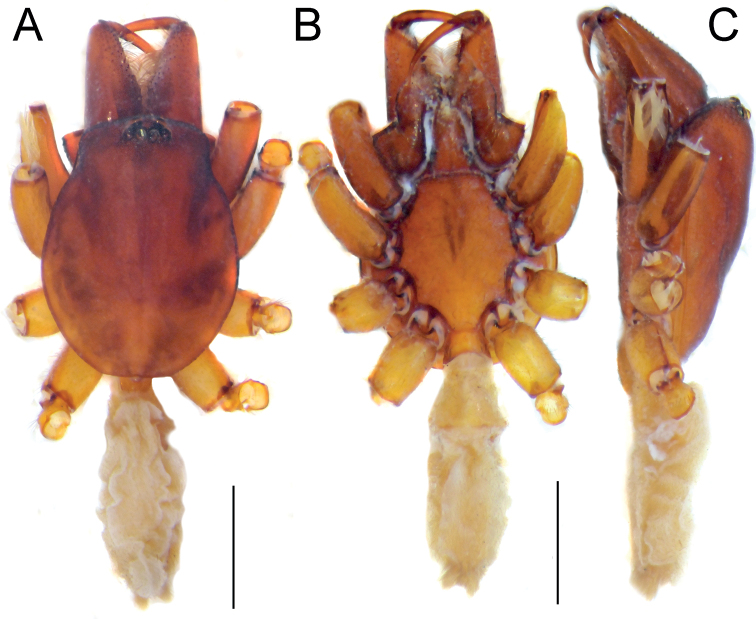
Male of *Dysderaxerxesi* sp. nov., habitus **A** dorsal view **B** ventral view **C** lateral view. Scale bars: 1.0 mm.

**Male.** Habitus as in Fig. 23A–C. Total length 4.02. Carapace 2.14 long, 1.60 wide. Eye diameters: AME 0.11, PME 0.09, PLE 0.08. Carapace, sternum, chelicerae, labium, and maxillae reddish brown. Legs missing. Abdomen cream-coloured, without any pattern. Spinnerets uniformly cream-coloured.

**Figure 24.**
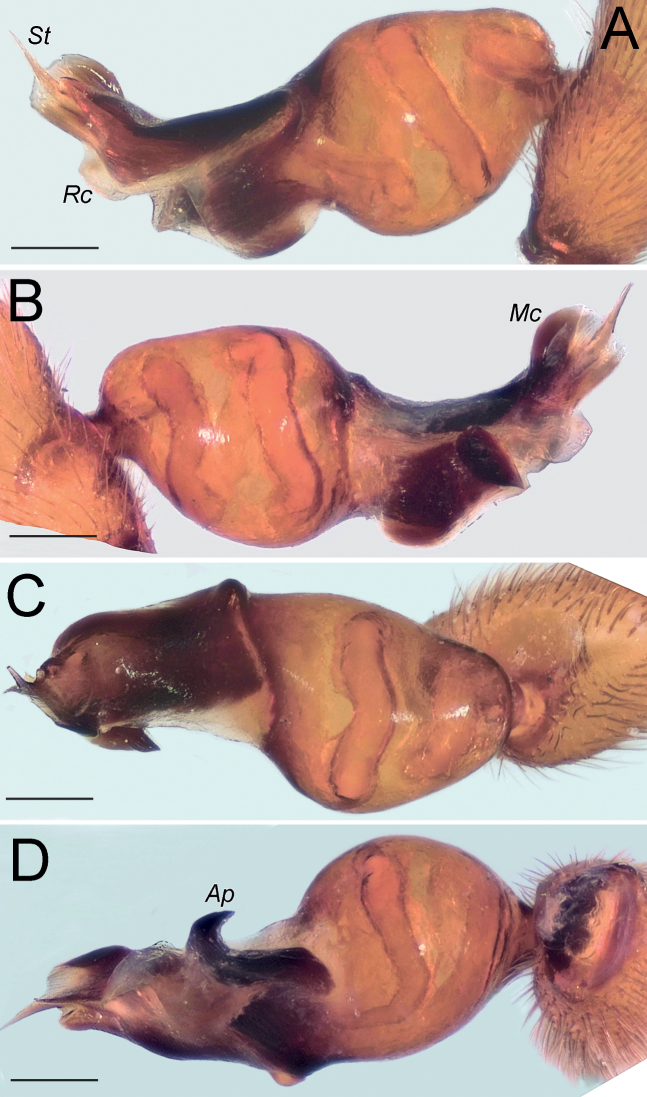
Male of *Dysderaxerxesi* sp. nov., bulb **A** retrolateral view **B** prolateral view **C** anterior view **D** posterior view. Scale bars: 0.25 mm. Abbreviations: *Ap* – posterior apophysis, *Mc* – median crest, *Rc* – retrolateral crest, *St* – stylus.

Palp as in Fig. 24A–D; bulb ca. 2.3× longer than wide; tegulum bell-shaped, almost as long as wide; psembolus 1.48× longer than tegulum; median crest (*Mc*) rounded, ca. 3.45× shorter than length of psembolus, ca. 2.3× wider than high; posterior apophysis (*Ap*) broad and hook-shaped; incision between tegulum and psembolus absent; retrolateral crest (*Rc*) gradually rounded; stylus (*St*) membranous and shorter than median crest.

**Female.** Unknown.

#### Distribution.

Known only from the type locality in Bushehr Province, southern Iran (Fig. 35).

##### ﻿*longirostris* species group

**Diagnosis.** This group can be diagnosed by a combination of the following characters: cheliceral fang as long as the basal segment, carapace broad, flat and anteriorly convergent, and bulb with lateral projection smaller than the apex (Deeleman-Reinhold and Deeleman 1988).

### ﻿ Dysdera damavandica sp. nov.

Taxon classificationAnimaliaAraneaeDysderidae

45DFBDC3-F666-5D64-AAF6-CC8B4DE06615

https://zoobank.org/4A417CC6-E9AD-4FA8-B3AD-750B3BB3184B

Figs 25A–C , 26A–D

#### Type material.

***Holotype*** ♂ (ZMUT), Iran: Mazandaran Province: Polur, surroundings of Mount Damavand, 35°50'N, 52°03'E, 10.2015 (A. Zamani).

#### Etymology.

The specific epithet is an adjective, referring to the type locality of the species.

#### Diagnosis.

The male of the new species is most similar to that of *D.concinna* L. Koch, 1878 from Azerbaijan, but differs by longer bulb (i.e., bulb length/tegulum width =3.1, vs. 2.7), relatively shorter median crest, and longer stylus (cf. Fig. 26A and Dunin 1982: fig. B). *Dysderadamavandica* sp. nov. is also similar to *D.tapuria* sp. nov. but differs by the median crest higher than wide (vs. wider than high), relatively longer stylus (cf. Fig. 26A and Fig. 30A) and posterior apophysis located at distal half of the bulb (vs. located at mid-part).

#### Description.

**Figure 25.**
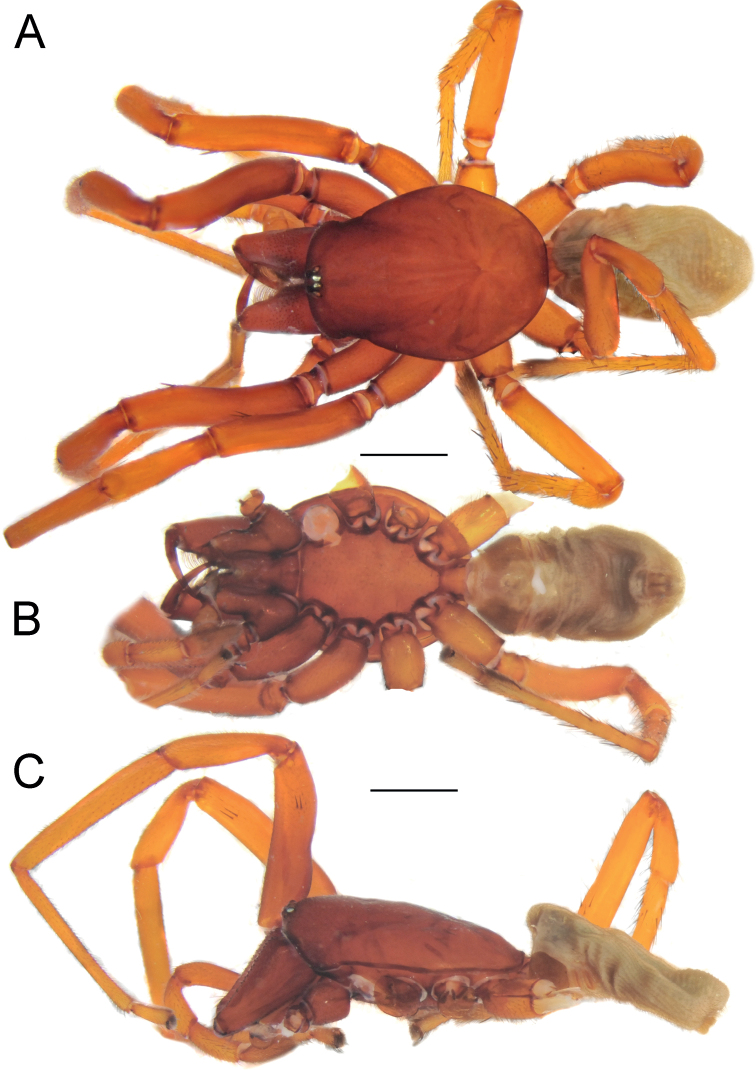
Male of *Dysderadamavandica* sp. nov., habitus **A** dorsal view **B** ventral view **C** lateral view. Scale bars: 1.0 mm.

**Male.** Habitus as in Fig. 25A–C. Total length 10.40. Carapace 5.53 long, 4.12 wide. Eye diameters: AME 0.18, PME 0.19, PLE 0.23. Carapace, sternum, chelicerae, labium, and maxillae reddish brown. Legs dark orange. Abdomen greyish, without any pattern. Spinnerets uniformly greyish. Measurements of legs: I: 15.64 (4.51, 2.54, 4.12, 3.56, 0.91), II: 15.12 (4.16, 2.67, 3.73, 3.69, 0.87), III: 10.91 (3.21, 1.73, 2.22, 3.01, 0.74), IV: 13.75 (3.82, 2.02, 3.13, 3.94, 0.84). Spination: I: Fe: 2pl. II: Fe: 2pl. III: Fe: 1pl; Ti: 4pl, 2rl, 5v; Mt: 3pl, 2rl, 3v. IV: Fe: 7d; Ti: 2pl, 3rl, 5v; Mt: 4pl, 3rl, 5v.

**Figure 26.**
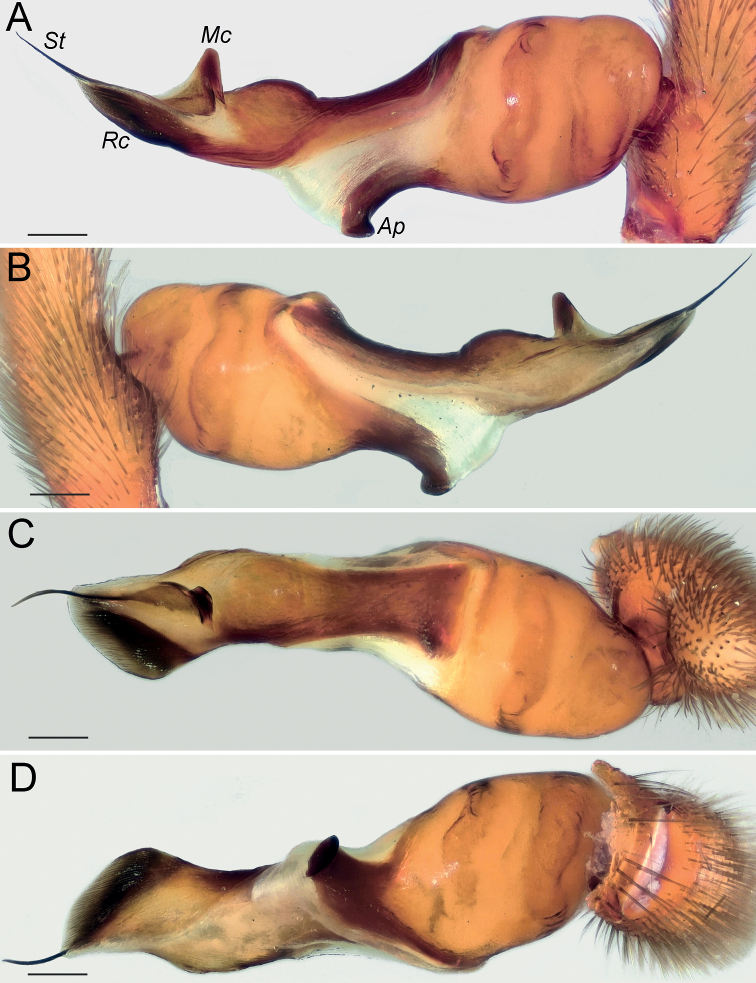
Male of *Dysderadamavandica* sp. nov., bulb **A** retrolateral view **B** prolateral view **C** anterior view **D** posterior view. Scale bars: 0.25 mm. Abbreviations: *Ap* – posterior apophysis, *Mc* – median crest, *Rc* – retrolateral crest, *St* – stylus.

Palp as in Fig. 26A–D; bulb ca. 3.1× longer than wide; tegulum bell-shaped, almost as long as wide; psembolus 2.77× longer than tegulum; median crest (*Mc*) triangular, ca. 6.65× shorter than length of psembolus, higher than wide; posterior apophysis (*Ap*) rounded; incision between tegulum and psembolus present; retrolateral crest almost straight; stylus (*St*) straight, longer than median crest.

**Female.** Unknown.

#### Distribution.

Known only from the type locality in Mazandaran Province, northern Iran (Fig. 35).

### ﻿ Dysdera medes sp. nov.

Taxon classificationAnimaliaAraneaeDysderidae

109EDCC1-C8CE-5091-8772-C0B4B0A10F88

https://zoobank.org/ED370972-D544-4411-A52E-4599D12E3850

Figs 27A–C , 28A–D

#### Type material.

***Holotype*** ♂ (ZMUT), Iran: Tehran Province: Tehran, 35°43'N, 51°24'E, 1994 (A. Savoji).

#### Etymology.

The specific epithet is a noun in apposition, referring to an ancient Iranian people who inhabited an area known as Media between western and northern Iran.

#### Diagnosis.

The male of the new species is similar to that of *D.granulata* Kulczyński, 1897 from Italy and the Balkan Peninsula, but differs by the shape of the tegulum (i.e., almost as wide as long, vs. 1.5× longer than wide), and by thinner psembolus (as wide as tegulum, vs. wider than tegulum). The male of *D.medes* sp. nov. differs from those of its congeners occurring in Iran by the very long median crest (i.e., longer than half of psembolus, vs. shorter), abrupt tip of psembolus in ventral and dorsal views (Fig. 28C, D) (vs. not abrupt), and posterior apophysis with two teeth (vs. one).

#### Description.

**Figure 27.**
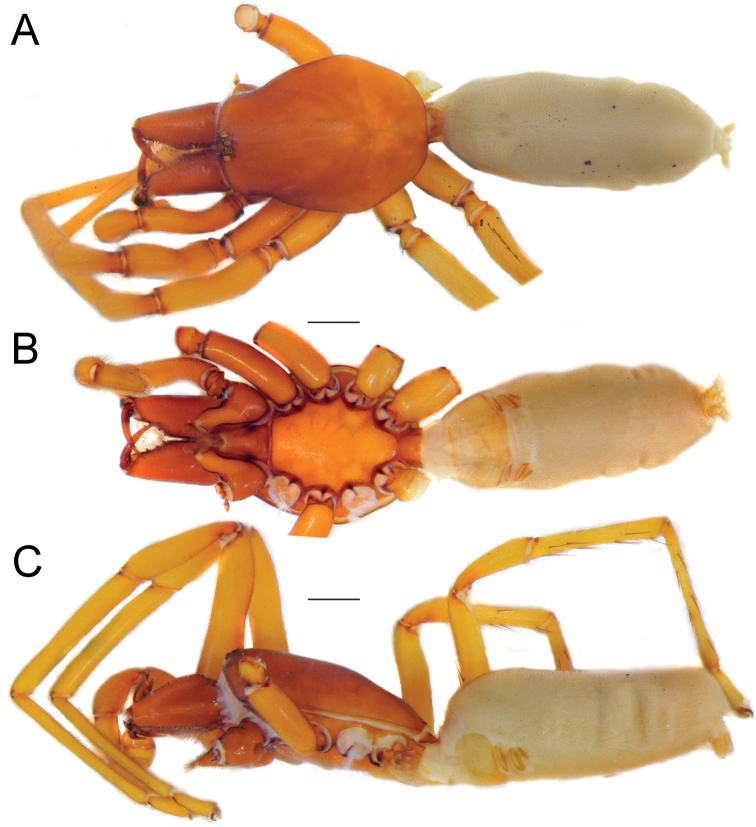
Male of *Dysderamedes* sp. nov., habitus **A** dorsal view **B** ventral view **C** lateral view. Scale bars: 1.0 mm.

**Male.** Habitus as in Fig. 27A–C. Total length 10.0. Carapace 4.19 long, 3.17 wide. Eye diameters: AME 0.14, PME 0.14, PLE 0.14. Carapace, sternum, chelicerae, labium, and maxillae reddish brown. Legs orange. Abdomen greyish, without any pattern. Spinnerets uniformly dark yellowish. Measurements of legs: I: 12.33 (3.54, 2.03, 2.89, 3.00, 0.87), II: 13.50 (3.80, 2.40, 3.12, 3.29, 0.89), III: 9.99 (2.95, 1.59, 1.86, 2.69, 0.90), IV: 12.31 (3.73, 1.80, 2.46, 3.30, 1.02). Spination: I, II: no spines. III: Ti: 4pl, 2rl, 5v; Mt: 6pl, 3rl, 2v. IV: Fe: 7d; Ti: 7pl, 4rl, 8v; Mt: 7pl, 3rl, 4v.

**Figure 28.**
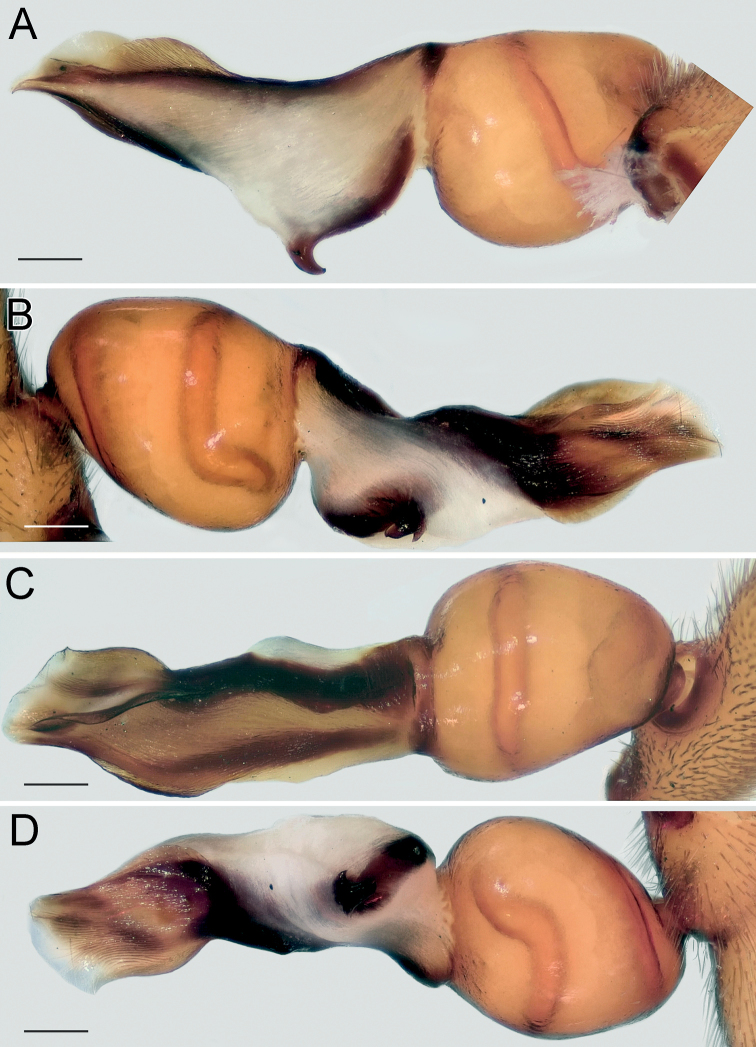
Male of *Dysderamedes* sp. nov., bulb **A** retrolateral view **B** prolateral view **C** anterior view **D** posterior view. Scale bars: 0.25 mm.

Palp as in Fig. 28A–D; bulb ca. 2.8× longer than wide; tegulum bell-shaped, almost as long as wide; psembolus 1.9× longer than tegulum; median crest rounded, ca. 2.42× shorter than length of psembolus, ca. 5.3× wider than high; posterior apophysis claw-shaped, with 2 teeth (Fig. 28B, D); incision between tegulum and psembolus absent; retrolateral crest almost straight.

**Female.** Unknown.

#### Distribution.

Known only from the type locality in Tehran Province, northern Iran (Fig. 35).

### ﻿ Dysdera tapuria sp. nov.

Taxon classificationAnimaliaAraneaeDysderidae

5C365310-7DE7-552F-8263-3A0D44AC1FB2

https://zoobank.org/67653B32-6B9D-494E-8921-C4D8CEDBE70B

**Figure 29.**
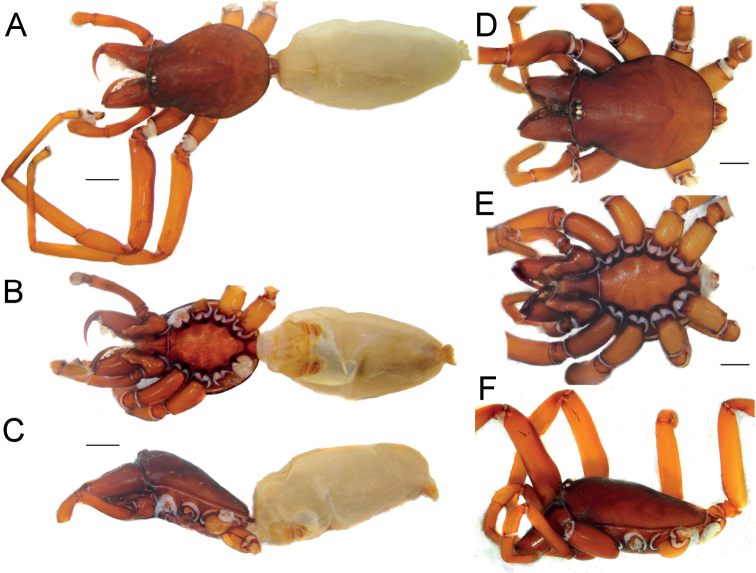
Male (**A–C**) and female (**D–F**) of *Dysderatapuria* sp. nov., habitus **A, D** dorsal view **B, E** ventral view **C, F** lateral view. Scale bars: 1.0 mm.

**Figure 30.**
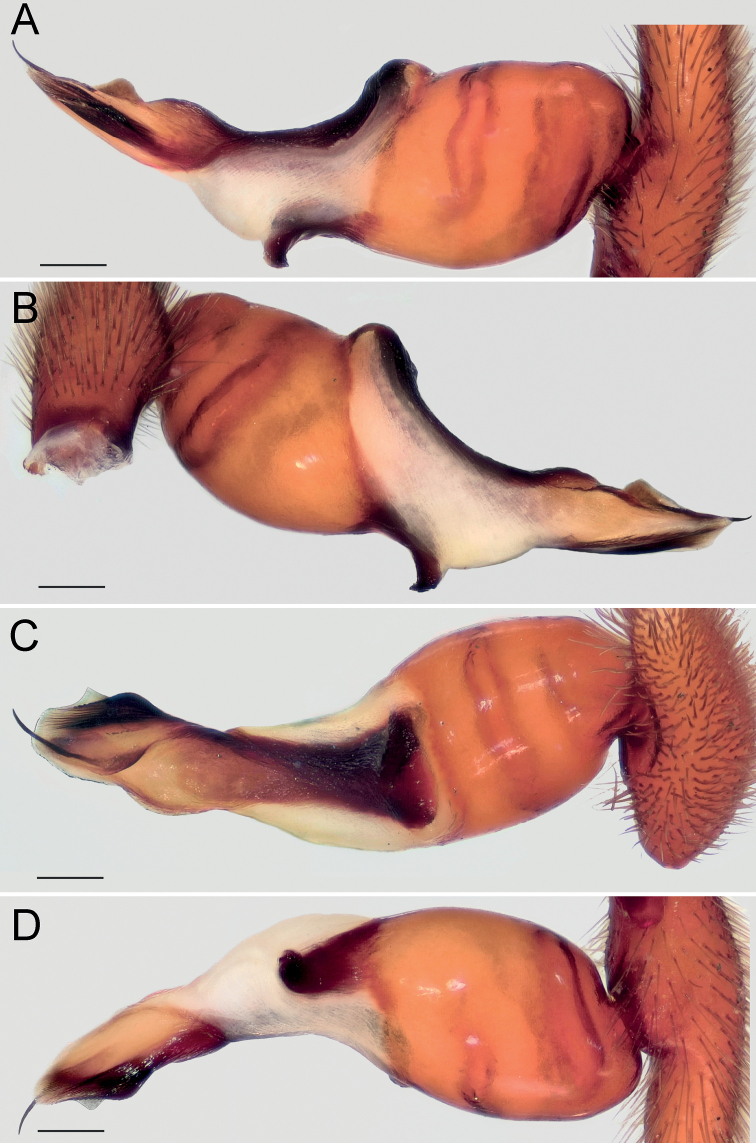
Male of *Dysderatapuria* sp. nov., bulb **A** retrolateral view **B** prolateral view **C** anterior view **D** posterior view. Scale bars: 0.25 mm.

**Figure 31.**
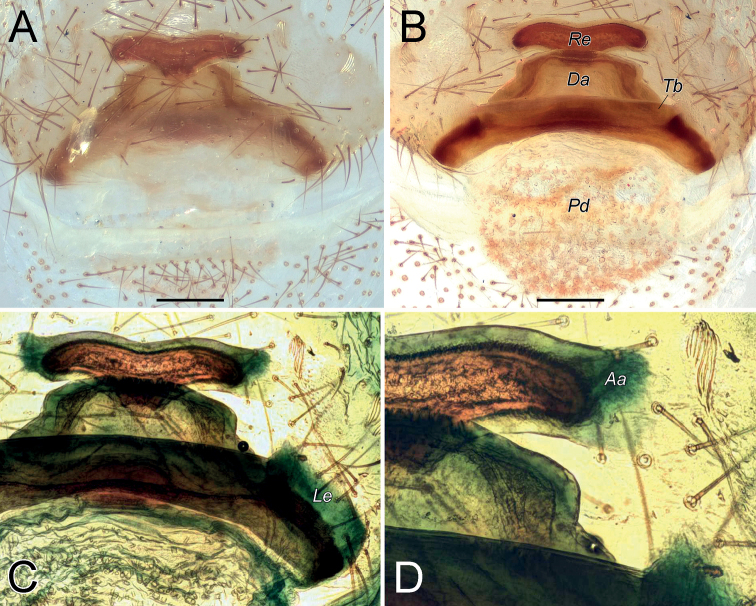
Female of *Dysderatapuria* sp. nov., endogyne **A** ventral view **B–D** dorsal view. Scale bars: 0.25 mm. Abbreviations: *Aa* – anterior angle, *Da* – dorsal arch, *Le* – lateral edge, *Pd* – posterior diverticulum, *Re* – receptacle, *Tb* – transverse bar.

Figs 29A–F , 30A–D , 31A–D

#### Type material.

***Holotype*** ♂ (MMUE), Iran: Mazandaran Province: Tooban, Khorram-Abad, 36°43'N, 50°48'E, 8–10.06.2000 (Y.M. Marusik). ***Paratypes***: 1♀ (MMUE), same data as the holotype; 1♂ (MHNG), Chorteh, 36°49'N, 50°38'E, 1300 m, 5.08.1974 (A. Senglet); 1♂ (MHNG), Chorteh, 36°49'N, 50°38'E, 1300 m, 8.07.1973 (A. Senglet).

#### Etymology.

The specific epithet is a noun in apposition, referring to the term applied to a mountainous region located in the Caspian coast of northern Iran.

#### Diagnosis.

The male of the new species is most similar to that of *D.concinna*, but differs by longer bulb (i.e., bulb length/tegulum width = 3.1, vs. 2.7), median crest wider than high (vs. higher than wide), and shorter stylus (cf. Fig. 30B and Dunin 1982: fig. B). The male of *D.tapuria* sp. nov. is also similar to that of *D.damavandica* sp. nov., but differs by the median crest wider than high (vs. higher than wide) and relatively shorter stylus (cf. Fig. 30A and Fig. 26A). The female of this species differs from those of its congeners occurring in the region by the very wide lateral edges of the receptacle (i.e., approximately half of the receptacle’s width, vs. less than half).

#### Description.

**Male (Holotype).** Habitus as in Fig. 29A–C. Total length 9.55. Carapace 3.76 long, 2.86 wide. Eye diameters: AME 0.14, PME 0.13, PLE 0.13. Carapace, sternum, chelicerae, labium, and maxillae reddish brown. Legs orange. Abdomen cream-coloured, without any pattern. Spinnerets uniformly dark yellowish. Measurements of legs: I: 14.04 (4.16, 2.14, 3.65, 3.24, 0.85), II: 12.72 (3.79, 2.08, 3.00, 3.11, 0.74), III: 10.01 (2.93, 1.44, 2.14, 2.77, 0.73), IV: 11.90 (3.72, 1.48, 2.73, 3.19, 0.78). Spination: I: Fe: 2pl. II: Fe: 1pl. III: Ti: 5pl, 3rl, 1v; Mt: 5pl, 2rl, 3v. IV: Fe: 8d, 1rl; Pa: 1pl; Ti: 5pl, 3rl, 6v; Mt: 6pl, 2rl, 5v.

Palp as in Fig. 30A–D; bulb ca. 3.1× longer than wide; tegulum bell-shaped, almost as long as wide; psembolus 1.8× longer than tegulum; median crest triangular, ca. 7× shorter than length of psembolus, ca. 2.5× wider than high; posterior apophysis claw-shaped; incision between tegulum and psembolus present; retrolateral crest roundly bent in proximal part and almost straight distally; stylus straight, as long as median crest.

**Female.** Habitus as in Fig. 29D–F. Total length 18.1. Carapace 7.77 long, 6.01 wide. Eye diameters: AME 0.30, PME 0.30, PLE 0.34. Colouration as in male. Measurements of legs: I: 14.24 (3.99, 2.54, 3.62, 3.44, 0.65), II: 13.46 (4.31, 2.10, 3.22, 3.06, 0.77), III: 10.20 (2.96, 1.70, 1.97, 2.75, 0.82), IV: 13.08 (3.99, 1.72, 2.85, 3.62, 0.90). Spination: I: Fe: 2pl. II: Fe: 2pl. III: Fe: 1pl; Ti: 2pl, 2rl, 4v; Mt: 3pl, 4rl, 5v. IV: Fe: 3d; Ti: 2pl, 2rl, 6v; Mt: 5pl, 4rl, 5v.

Endogyne as in Fig. 31A–D; length/width ratio ca. 3; receptacle (*Re*) with shallow median concavity, ca. 4.5× longer than wide, anterior angles (*Aa*) indistinct; dorsal arch (*Da*) trapezoidal, posterior margin ca. 1.6× longer than anterior, anterior angles rounded; transverse bar (*Tb*) almost 2× longer and slightly wider than receptacle; transverse bar’s anterior margin straight, posterior margin arched; lateral edges (*Le*) very wide, directed latero-posteriorly; posterior diverticulum (*Pd*) rounded.

#### Distribution.

Known only from the listed localities in Mazandaran Province, northern Iran (Fig. 35).

##### ﻿*ninnii* species group

**Diagnosis.** This group can be diagnosed by a combination of the following characters: chelicerae shorter than the width of carapace, carapace relatively short with anteriorly converging lateral margins, and bulb with simple crest, simple apex bearing a long subapical tooth, and a crescent-shaped lateral projection (Deeleman-Reinhold and Deeleman 1988).

### ﻿ Dysdera genoensis sp. nov.

Taxon classificationAnimaliaAraneaeDysderidae

DF2FB01E-BB6C-53BD-92AB-A606E75F0616

https://zoobank.org/0B0CBC7E-F121-4D54-BC35-364827CB6ECC

**Figure 32.**
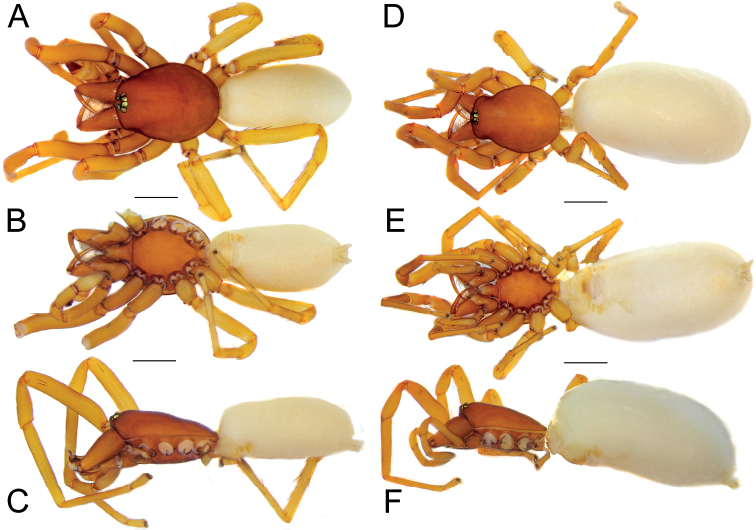
Male (**A–C**) and female (**D–F**) of *Dysderagenoensis* sp. nov., habitus **A, D** dorsal view **B, E** ventral view **C, F** lateral view. Scale bars: 1.0 mm.

**Figure 33.**
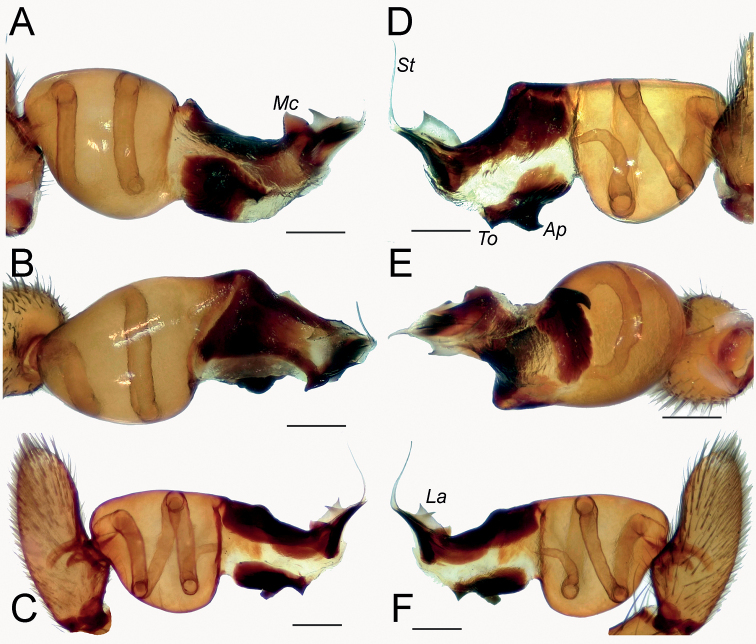
Male of *Dysderagenoensis* sp. nov., bulb **A** proanterior view **B** anterior view **C** prolateral view **D** retroposterior view **E** posterior view **F** retrolateral view. Scale bars: 0.25 mm. Abbreviations: *Ap* – posterior apophysis, *La* – laminae, *Mc* – median crest, *St* – stylus, *To* – triangular outgrowth.

**Figure 34.**
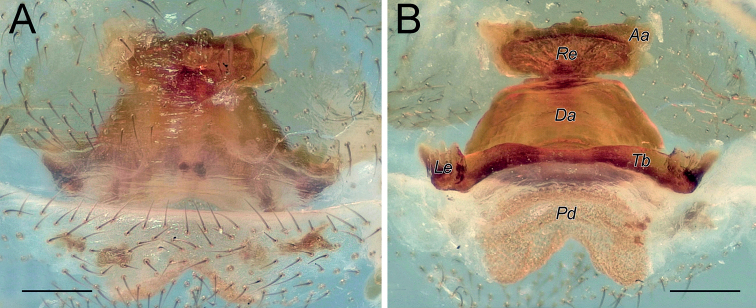
Female of *Dysderagenoensis* sp. nov., endogyne **A** ventral view **B** dorsal view. Scale bars: 0.25 mm. Abbreviations: *Aa* – anterior angle, *Da* – dorsal arch, *Le* – lateral edge, *Pd* – posterior diverticulum, *Re* – receptacle, *Tb* – transverse bar.

Figs 32A–F , 33A–F , 34A, B

#### Type material.

***Holotype*** ♂ (ZMUT), Iran: Hormozgan Province: Geno Biosphere Reserve, 27°22'N, 56°07'E, 02.2020 (A. Zamani). ***Paratypes***: 3♀ (ZMUT), same data as the holotype.

#### Etymology.

The specific epithet is an adjective, referring to the type locality of the species.

#### Diagnosis.

The male of the new species differs from those of its congeners by having weakly sclerotized bent stylus (*St*) (Fig. 32C, F) (vs. stylus, if present, not bent). The female of this species is very similar to that of *D.iranica* sp. nov. by having a wide receptacle (i.e., > 2× wider than transverse bar), but differs by the relatively wider receptacle lacking an anterior triangular projection, and the presence of a median concavity on the transverse bar (vs. receptacle with triangular projection, transverse bar without concavity).

#### Description.

**Male (Holotype).** Habitus as in Fig. 32A–C. Total length 5.49. Carapace 2.42 long, 1.88 wide. Eye diameters: AME 0.15, PME 0.13, PLE 0.11. Carapace, sternum, chelicerae, labium, and maxillae pale reddish. Legs orange. Abdomen cream-coloured, without any pattern. Spinnerets uniformly cream-coloured. Measurements of legs: I: 11.78 (3.23, 1.90, 2.87, 3.02, 0.76), II: 10.21 (2.91, 1.60, 2.52, 2.45, 0.73), III: 7.98 (2.31, 1.15, 1.65, 2.29, 0.58), IV: 10.30 (2.89, 1.44, 2.28, 2.96, 0.73). Spination: I: Fe: 2pl. II: Fe: 1pl. III: Fe: 1pl; Ti: 3pl, 2v; Mt: 3pl, 4rl, 3v. IV: Fe: 4d; Pa: 1pl; Ti: 3pl, 1rl, 4v; Mt: 1pl, 1rl, 3v.

Palp as in Fig. 33A–F; bulb ca. 2.3× longer than wide; tegulum bell-shaped, almost as long as wide; psembolus as long as tegulum, length/width ratio ca. 1.25; median crest (*Mc*) triangular; apex with two triangular laminae (*La*); posterior apophysis (*Ap*) claw-shaped, accompanied by a triangular outgrowth (*To*) anteriorly; incision between tegulum and psembolus absent; stylus (*St*) bent, long, and weakly sclerotized.

**Female.** Habitus as in Fig. 32D–F. Total length 6.52. Carapace 2.06 long, 1.59 wide. Eye diameters: AME 0.11, PME 0.11, PLE 0.10. Colouration as in male. Measurements of legs: I: 7.71 (1.93, 1.37, 2.01, 1.88, 0.52), II: 7.31 (1.99, 1.26, 1.77, 1.74, 0.55), III: 5.84 (1.58, 0.92, 1.19, 1.65, 0.50), IV: 7.77 (2.19, 1.21, 1.79, 2.04, 0.54). Spination: I: Fe: 2pl. II: Fe: 2pl. III: Fe: 1pl; Ti: 2pl, 1rl, 5v; Mt: 5pl, 2rl. IV: Fe: 5d, 1rl; Pa: 1rl, 1v; Ti: 5pl, 1rl, 5v; Mt: 4pl, 2rl, 3v.

Endogyne as in Fig. 34A, B; length/width ratio ca. 2; receptacle (*Re*) inverted trapezoidal, anterior margin 1.4× longer than posterior; dorsal arch (*Da*) trapezoidal, posterior margin 1.6× longer than anterior margin, 1.25× longer than receptacle; anterior margin of transverse bar (*Tb*) with median concavity, posterior margin arched; lateral edges (*Le*) small, approximately as long as wide; posterior diverticulum (*Pd*) bilobed.

#### Distribution.

**Figure 35.**
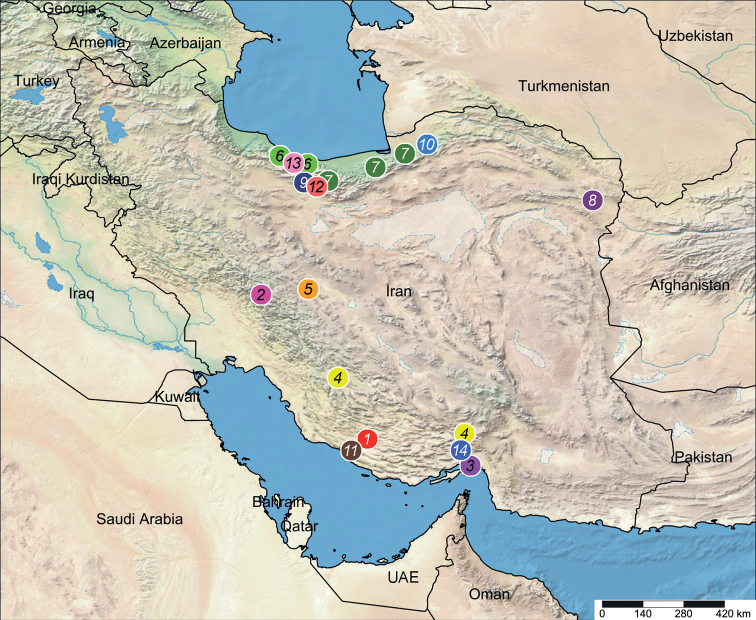
Distribution records of *Dysdera* spp. in Iran **1***D.achaemenes* sp. nov. **2***D.bakhtiari* sp. nov. **3***D.hormuzensis* sp. nov. **4***D.iranica* sp. nov. **5***D.isfahanica* sp. nov. **6***D.mazeruni* sp. nov. **7***D.persica* sp. nov. **8***D.pococki***9***D.sagartia* sp. nov. and *D.medes* sp. nov. **10***D.verkana* sp. nov. **11***D.xerxesi* sp. nov. **12***D.damavandica* sp. nov. **13***D.tapuria* sp. nov. **14***D.genoensis* sp. nov.

Known only from the type locality in Hormozgan Province, southern Iran (Fig. 35).

## ﻿Discussion

Considering the results of this paper, there are 17 species of three genera of Dysderidae known from Iran. This number is considerably lower than what is known for the neighbouring Turkey (i.e., 69 species in seven genera; Danışman et al. 2022) and the Caucasus (i.e., 65 species in seven genera; Otto 2022). The material treated here, although comprising a relatively large number of new species, were collected in a few localities primarily in northern parts of the country (Fig. 35). Considering the small distribution ranges of most dysderids and the presence of several mountainous regions and biodiversity hotspots in Iran (e.g., Alborz and Zagros mountain ranges; Farashi and Shariati 2017), it can be assumed that any new material from this region could potentially comprise further undescribed species. It is possible that the true diversity of Dysderidae in Iran could range between 40 to 60 species, if not higher; interestingly, the widespread and cosmopolitan *D.crocata* has not yet been recorded from this country.

Furthermore, the diversity of Dysderidae in Central Asia is relatively low (i.e., 21 species of three genera; Mikhailov 2022). Most of the species (18) belong to *Dysdera*, and *Dysderella* and *Harpactea* are known only from western Turkmenistan (Dunin 1992; Zamani et al. 2017). The eastern boundary of the family is westernmost Xinjiang, and it appears that their diversity gradually decreases east of the Caucasus (Fomichev and Marusik 2021).

## Supplementary Material

XML Treatment for Dysderidae

XML Treatment for Dysderinae

XML Treatment for Dysdera

XML Treatment for Dysdera achaemenes

XML Treatment for Dysdera bakhtiari

XML Treatment for Dysdera hormuzensis

XML Treatment for Dysdera iranica

XML Treatment for Dysdera isfahanica

XML Treatment for Dysdera mazeruni

XML Treatment for Dysdera persica

XML Treatment for Dysdera pococki

XML Treatment for Dysdera sagartia

XML Treatment for Dysdera verkana

XML Treatment for Dysdera xerxesi

XML Treatment for Dysdera damavandica

XML Treatment for Dysdera medes

XML Treatment for Dysdera tapuria

XML Treatment for Dysdera genoensis
